# Ethics in genetic counselling

**DOI:** 10.1007/s12687-018-0371-7

**Published:** 2018-06-14

**Authors:** Angus J. Clarke, Carina Wallgren-Pettersson

**Affiliations:** 1 Institute of Medical Genetics, Division of Cancer & Genetics, School of Medicine, Cardiff University, Heath Park, Cardiff, Wales CF14 4XN UK; 2 The Folkhaelsan Department of Medical Genetics, Topeliusgatan, 20 00250 Helsinki, Finland; 3 The Folkhaelsan Institute of Genetics and the Department of Medical and Clinical Genetics, University of Helsinki, Helsinki, Finland

**Keywords:** Ethics, Genetic information, Consent, Disclosure, Non-directiveness, Predictive genetic testing, Prenatal diagnosis, Disability, Screening, Incidental findings, Additional findings, Variants of uncertain significance

## Abstract

Difficult ethical issues arise for patients and professionals in medical genetics, and often relate to the patient’s family or their social context. Tackling these issues requires sensitivity to nuances of communication and a commitment to clarity and consistency. It also benefits from an awareness of different approaches to ethical theory. Many of the ethical problems encountered in genetics relate to tensions between the wishes or interests of different people, sometimes even people who do not (yet) exist or exist as embryos, either in an established pregnancy or in vitro. Concern for the long-term welfare of a child or young person, or possible future children, or for other members of the family, may lead to tensions felt by the patient (client) in genetic counselling. Differences in perspective may also arise between the patient and professional when the latter recommends disclosure of information to relatives and the patient finds that too difficult, or when the professional considers the genetic testing of a child, sought by parents, to be inappropriate. The expectations of a patient’s community may also lead to the differences in perspective between patient and counsellor. Recent developments of genetic technology permit genome-wide investigations. These have generated additional and more complex data that amplify and exacerbate some pre-existing ethical problems, including those presented by incidental (additional sought *and* secondary) findings and the recognition of variants currently of uncertain significance, so that reports of genomic investigations may often be provisional rather than definitive. Experience is being gained with these problems but substantial challenges are likely to persist in the long term.

## Introduction

This paper outlines the issues and the material discussed in a module on “ethical issues in genetic counselling” that is a key feature of the Cardiff University MSc course in Genetic Counselling. It cannot be complete and exhaustive and focuses on genetic counselling practice in UK and Europe, but we hope it may be of interest also to those who work elsewhere.

### Reflecting on the past

Ethics is important in the practice of every area of medicine, but this is nowhere more true than in attending to the genetic aspects of disease. Why is this so?

In part, this arises from the history of genetics. Public discussion about the inheritance of disease and non-disease traits in humans began in the nineteenth century England, led by the polymath Francis Galton. From its beginning, it was associated with attempts to improve society through selective breeding, as if mankind was another farmyard animal with some strains worthy of encouragement and others not. The discoveries of Mendel in his investigation of plants had not then been widely recognised and Galton approached inheritance predominantly from a population perspective rather than the more focused clinical perspective of the individual. Galton’s approach led to attempts to improve the genetic health of the population, which he termed ‘eugenics’. This population perspective was taken up by philanthropists and politicians and led to the ideas of positive eugenics (encouraging some to breed) and negative eugenics (discouraging others). Such ideas were seen as eminently respectable across Europe and North America at the turn of the 19th into the twentieth century. The forced sterilisation of those deemed unfit to reproduce followed from this logic in many countries, including Sweden and USA. In Nazi Germany, this approach was developed further into a policy of killing those patients considered unfit to live. The medical profession and associated scientists, especially anthropologists, often colluded with these activities in a devastating Faustian pact.

These events occurred in societies that viewed populations as both inherently distinct and of unequal value. Ideas about a natural heirarchy of racial types coalesced with ideas about the incipent degeneration of any population if the less worthy types were not stopped from breeding to excess. The effort to maintain the purity and quality of the race—racial hygiene—became especially closely entangled in Nazi ideology with their parallel beliefs about racial superiority, leading to the murder of much larger numbers of those deemed inferior because of their assigned race. However, what is common to the two activities is the assessment of the worth of an individual from a population perspective: what good to the nation or the state is this individual with a malformation or a cognitive impairment? Of what use is this man or woman from an ‘inferior race’?

One use that could be made of inferior types in Nazi Germany was a role in human experimentation: the name of Mengele remains infamous to this day. Some of his medical colleagues gained high repute from their pathological studies conducted post mortem on their patients, whom they had selected to be killed for this purpose (Müller-Hill 1988; Harper 1996).

Apartheid can be seen as another application of this ideology, with the mixing of ‘races’ seen as a contamination that would lead to a degeneration of the true stock. The language of genetics could just as easily have promoted such mixing of populations through citing the benefits of ‘hybrid vigour’ but that perspective is not appealing to the eugenicists.

The central lesson for us from this experience must be that any benefits to a population or nation from clinical genetic services should arise only as a secondary consequence of the primary benefits to patients and their families. Benefits to the population should never be used as the principal goal of clinical genetic services, which should always give priority to the interests and wishes of the individuals involved. Similarly, any experimentation or research involving humans must be conducted with the consent of the research participants, who must be fully aware of any potential risks.

Since the end of World War II, the medical profession has largely accepted these lessons, drawn from reflection on the abuse of patients under the Nazi regime, and they are applied far beyond the scope of genetics. Thus, the requirement of informed consent for research is a central principle of human rights under the terms of the World Medical Association’s Declaration of Helsinki in 1964 (updated most recently in 2017). We are reminded of the need for this when we consider some of the flagrant counter examples, such as the Tuskegee syphilis study, which abused research subjects for a decade after ‘Helsinki’. Despite these grounds for caution, enthusiasts for population screening programmes have sometimes strayed close to the eugenic motivation of the Nazi race hygienists, justifying their interventions by the reduced birth rate of children born with malformation or cognitive impairment and/or the financial savings to the state. Indeed, it is sometimes unclear whether the motivation of enthusiasts for antenatal screening is the ‘health’ of the population, the promotion of the informed choices of parents, concern about the financial pressures on the state or (sometimes) their own personal financial gain through their role in providing screening tests. Conflicts of interest abound, and mixed motives may apply. It is to be noted that according to the 2017 update of the Declaration, the Physician explicitly pledges not to use her medical knowledge to violate human rights and civil liberties, even under threat.

While few western governments would now espouse an explicitly eugenic approach to genetic population health, the underlying motivation for some antenatal screening programmes may be very ‘traditional’ in this respect, despite a veneer of politically correct rhetoric. In a time of financial austerity, and with fading confidence in the equitable, state-led provision of health care, social care and special education for those with physical and/or cognitive impairment, the choices being made by families in antenatal screening are not made in a vacuum. Their decisions may be their own, but they may be tightly constrained by circumstance and will not always reflect their own wishes and values.

### Reason, emotion and experience

Another reason for those who work in clinical genetics to consider the ethics of their profession is that this topic area engages us all, both patients and professionals, in a very deep and personal sense. We are engaged in our heads and in our hearts, both rationally/cognitively and emotionally, and the two do not always mesh well together. It is possible to make a decision objectively, in a cognitively ‘detached’ fashion, and then find that it conflicts with one’s feelings. A logical process of reasoning may lead to a decision that one finds unacceptable or even repugnant. Our task is to help our patients understand their position biologically but then make personal decisions that they can live with in the long term. The decisions must be theirs, but we can help them to consider the various factors that may be relevant, including factors they had not thought of. The potential for a gulf between a detached and objective decision and one’s feelings will often arise in decisions about the sharing of information within the family, about predictive genetic testing where there are no available treatments, and about prenatal genetic testing where the only medical intervention available would be a termination of pregnancy.

The assessment of the value of our work is much more complex and nuanced than in many other areas of medicine. Whereas there may be simple and objective measures of outcome for the success of an orthopaedic procedure or the treatment of organ failure, our criteria for the success of a consultation are set by the patient and, therefore, will vary from one consultation to the next, even for the same condition. We have to ask our patients what they want from a consultation and then do our best to achieve it. It is so patient-led that measures of outcome, that would be valid across consultations and conditions and between patients, are difficult to develop. The most satisfactory measure of outcome is one based on the concept of patient empowerment (McAllister et al. 2011).

In practice, we have to ask each patient about their particular concerns and decide with them what we can do to support them. The core activity of clinical genetics and genetic counselling is therefore communication, most especially listening to our patients. However, we may then have to help our patients reassess their situation and revise their decisions in the light of an enhanced understanding of ‘the facts’ and also of their social and family context. So, we need to listen, to assess the biological facts as far as we are able, to provide the relevant ‘medical’ information to the patient and family in terms that can be digested and applied by them, and then help them adjust to their situation, so that they make decisions that are true to their nature while grounded in as accurate an understanding of the biological facts as possible.

Our support may involve helping a patient come to a decision in the light of both ‘the facts of the matter’ and of an awareness of their own likely response to the situation, which will combine cognitive, rational elements with sometimes powerful emotions. The patient has to integrate the facts with their feelings.

The term ‘non-directiveness’ is often used to convey the ethos of the profession, but this can be misleading: it may suggest that we merely provide information and then leave decisions to the patient. If true, that would, as we discuss further below, be a superficial approach to genetic counselling, akin to the abandonment of our patients when they most need our support. We would propose an active and engaged form of non-directiveness, in which it may be our role to challenge our patients, asking them to consider important factors that they may have chosen to avoid. We will ask them to consider a number of ‘what if ….?’ scenarios, so that they do not undertake a genetic test without considering the full range of options in front of them (including a decision not to have the test) and the full range of potential outcomes of testing (including an unclear result or an important but unanticipated finding, incidental to the reason for having the test).

### A framework for ethics in the clinical genetics consultation

The first element of ethics is to recognise a problem as being ethical, i.e. that there is a question about what one ought to do. This may appear to be obvious but there are several ways in which the obvious can be obscured. First, it may appear that the ethical aspect of a situation—what ought to happen—applies to someone else, such as the patient or a member of their family or perhaps society’s arrangements for funding health care. While the political context may on occasion be relevant to the problems arising within the clinic, especially if resources are limited, and one may feel obligations as a citizen to address such questions, this does not resolve the ethical issues that still face us, day to day, in the meantime.

Secondly, problematic aspects of genetic consultations may be framed as ethical (for the patient or for the professional) but may also be open to framing in other ways, perhaps as an issue of counselling practice or as a question of the social consequences of a patient’s decision for others in their family, rather than a question of professional ethics. It may be possible to frame the same problem in any or all of these ways.

Having recognised the ‘ought’ element in the setting, especially as it applies to oneself (the professional), one then needs a framework through which to approach it. We need to consider who is involved and what their interests are (or might be). This could entail considering the interests of an unborn child or of future people (not yet conceived), whose interests could not be considered in a court of law but who must be considered from the broader, more inclusive perspective of ethics. Listing those involved and their potential interests can be helpful in clarifying what is at stake and for whom.

The third element of this framework is to consider potential solutions, noting whose interests they take fully and fairly into account. Then, the final element is to formulate a coherent argument about which of these potential solutions to work towards (i.e. making a decision about what to do).

This fourfold framework may seem rather insubstantial, but it is a helpful approach in addressing even the most complex problems. It is not a theory in its own right, however, and it will need to be supplemented by other insights drawn from logic and moral philosophy. In describing the ethical aspects of a problem and in formulating an argument, it is essential that any underlying assumptions are explicit and that the steps in the process of reasoning are clear and valid (Flew 1975; Holm 2004): we will not go far into this here, but it is important to consider the consistency of an argument as it comes to be applied to similar but non-identical instances, and to approach any explicit discussion of ethics with patients with great care (Kaldjian 2013) as such discussions could easily become manipulative. A broad notion of the other ‘interested parties’ whose interests are to be considered must be used, so that the opportunity costs of a course of action are included in the assessment, along with wider issues of social justice. And the steps that may be needed to resolve a difference of opinion between individuals or groups—the procedures involved—may need to be made explicit, as in Habermas’s discourse ethics. Such an approach places emphasis on the value of following a process or procedure impartially and with consistency.

An appreciation of the major schools of moral philosophy is very helpful (Blackburn 2001). Those who examine questions of ethics from a specific philosophical or theological position may adopt different approaches to thinking through these questions, so that some familiarity with important schools of thought may be useful.

Consequentialism assesses a course of action by weighing the outcomes it leads to, the best known consequentialist approach being utilitarianism. Deontology draws on rules, including ethical principles, rights and obligations, to determine the right course of action. Virtue-based ethics is grounded in ideas of human nature and the ends that we should pursue, individually and collectively within society. If we do not maintain this broader awareness of ethical theory, it becomes all too easy for medical ethics to reduce to the mechanical application, as if by rote, of the well-known Four Principles set out by Beauchamp and Childress (autonomy, beneficence, non-maleficence and justice) (Beauchamp and Childress 2013).

In considering eugenics, as above, one could say that Galton’s eugenics is utilitarian in its attempt to maximise the overall welfare (or happiness) of society. Objections to eugenics will often be Kantian, as Kant was famous for his categorical imperative, one formulation of which specifies that a person should never use other people as mere means to her own chosen end(s).

What is needed to address the ethical issues is a full engagement of the professional as a person on every level: the rational and intellectual, indeed, but also the interpersonal awareness needed to understand the perspectives of others and to recognise the subtleties of meaning that may be expressed obliquely in communication, and the creative imagination to consider likely emotional responses in different scenarios and to generate a range of possible solutions. A good place to start practising these skills can be the consideration of ‘a case’ from the perspective of those most closely involved (e.g. Thiele 2010; Wilkinson 2010).

In the rest of this chapter, we turn to consider the major, recurrent issues that arise in genetic counselling practice and, more generally, in medicine as it deals with genetic disorders. A fuller account of these areas is given by Parker (2012) and a survey of the issues emerging as genomic technologies enter clinical practice is given in Clarke (2014).

## Family matters: secrecy, disclosure, communication and testing

A recurrent question in genetic counselling practice is, ‘When—and how—should we help, encourage and perhaps persuade patients to share their personal medical and genetic information with relatives and those close to them?’ Important medical information about one person may be relevant to their family members for many reasons. If one person in a family is found to be at risk of developing a cancer or a serious cardiac problem, for example, then medical surveillance may be helpful to reduce morbidity and/or mortality in others as well as in them. A relative may also want to know if they are at risk of developing a late-onset neurodegenerative condition, such as Huntington’s disease, or if their children may be at risk of a serious disease or a genetic condition that can cause a disease, a cognitive or sensory impairment or a malformation that has already affected one or more members of the family.

Of course, such information, when it may be relevant to family members and when this is explained to the patient, will usually be passed to relatives. This is especially true if the unaffected but at-risk relatives can take action to avoid at least some of the likely problems. They may be able to have screening for malignancies of the breast or bowel, for example, or for a disorder of cardiac muscle or rhythm, or they may watch for a potentially silent complication such as hypertension at an unusually early age, as in polycystic kidney disease or neurofibromatosis type 1. Awareness of such problems can substantially improve the person’s likely prognosis.

Passing information to relatives can be experienced as more difficult if there are no interventions known to improve the outlook for those who have inherited the condition; this applies (at least for now) in families affected by Huntington disease (HD) and other neurodegenerative disorders. Telling a relative that they may be at risk of such a condition but that there is no treatment, so that ‘nothing can be done about it’, may be a thankless task, with the relatives possibly becoming angry, frightened and resentful. The family informant may therefore decide to wait ‘until the time is right’ before passing on the unwelcome information. But there may never be a ‘right’ time. Family gatherings may provide an opportunity for such discussions, but it may feel inappropriate to raise such questions at a wedding, or a funeral, or at Christmas. The disclosure of genetic information by an at-risk adult to their partner, before marriage or reproduction, can also be a very difficult decision (Keenan et al. 2013).

Conversely, when there is a helpful intervention, the motivation to pass on information is stronger. Families are often willing to let health services make contact directly with their relatives when the medical benefit is clear, as in familial hypercholesterolaemia (Newson and Humphries 2005). On the other hand, when such a condition is not seen as very strongly inherited but rather as a routine question for anyone of managing their own cholesterol level, the drive to pass information to relatives may be less (Weiner 2011).

There have been numerous studies, often conducted by social scientists, of communication about difficult genetic information within families. Such studies can be referred to as research in ‘empirical ethics’ as the findings help ethicists to remain grounded in their discussions in the concrete and often messy reality of everyday life. Important and helpful studies of this type are presented by Forrest et al. (2003) and Featherstone et al. (2006). They discuss the barriers that can obstruct the passing of information, such as geographical distance, family estrangement, concern about the emotional readiness of a relative to be given unwelcome information and concern about the possible emotional response of the individual, who may become distressed or angry. Professionals can draw on this work to help their patients plan how best to disclose information to relatives. These studies remind professionals of the difference between ‘giving’ information and ‘receiving’ it: one member of a family may consider that they have passed on the information but the ‘recipient’ may never have ‘received’ it. Important information should be given in a very clear and explicit fashion, and it may be helpful for professionals to back this up with written information such as letters ‘To Whom It May Concern’ that can be passed by patients to their relatives.

The studies of both Forrest and Featherstone explore the dynamics of disclosure within families, showing how they are shaped by many factors including gender roles, with women often assuming the management of information within a family, even when the relevant inheritance relates to their partner’s family (d’Agincourt-Canning 2001). It is difficult to address these factors realistically without colluding with the (western) stereotypes of the silent male and the busybody female. However, what is crucial for the clinician is to make no assumptions about how the particular individuals in a family will be inclined to behave. The importance of an accurate understanding of inheritance cannot be over-emphasised, especially when gender roles and the biological facts combine to downplay the involvement of males, so that the family may fail to appreciate that fathers can transmit a risk of breast cancer to their daughters (Hallowell et al. 2006).

Even testing for carrier status in a family affected by an autosomal recessive disorder may have personal repercussions for family members, both the affected individual and the unaffected but possibly carrier siblings. Testing for carrier status puts at risk the sense of family unity that is triggered by the disease in question. A choice to be tested may suggest disloyalty to the affected family member(s), as by implication it suggests that the patient’s brother or sister would not want to have a child like their affected sibling, while finding that one is not a carrier may lead the healthy sibling to feel isolated from the family, as no longer involved with the disease in the way that the others are (Fanos and Johnson 1995a, b).

The feelings raised by carrier testing in the context of sex-linked disorders and chromosome rearrangements may be rather stronger for two reasons: that the ‘carrier’ may sometimes be affected, at least to some extent, and that the condition is transmitted to the child solely by them, although the decision to have children will usually be a shared decision with their partner. In terms of the weight or burden of being a carrier, carrier status for these disorders may be seen as intermediate between the situation in autosomal dominant and autosomal recessive disorders.

Genetics health professionals usually handle difficult family situations in practice by maintaining links with the patient and attempting to persuade them to disclose the relevant information to their relatives. It is exceptional for professionals to force disclosure of information about one person to another against their wishes (Clarke et al. 2005). There are important procedures to follow before a practitioner should implement such a disclosure, differing between jurisdictions. In UK, the General Medical Council has issued guidance on this. Recent changes to the law in France have emphasised the role of the family member in passing on information but this may then leave an obligation on the professional to ensure that this has happened.

A particular issue faced occasionally by health professionals is that of misattributed paternity that comes to light through genetic testing. While the ‘abstract’ ethical arguments weigh heavily in favour of disclosing this to the ‘social father’ as well as to the mother in a couple who have come for testing (Lucassen and Parker 2001), in practice professionals usually try to avoid precipitating confrontation and conflict within a couple. One lesson from such experiences is to make clear in advance to those involved when a genetic test is being discussed that might reveal misattributed paternity. This issue can also arise when the daughter of a man with a sex-linked disorder requests confirmation of her obligate carrier status.

In addition to gender, ‘culture’ may be seen as an important factor in family communication within some communities. If consanguinity is customary within a community, and if carrier status for a genetic disorder may attract stigma and lead to difficulty in arranging marriage for oneself or one’s kin, a family may wish to conceal their genetic condition from everyone else, and especially from other members of the family (Shaw and Hurst 2009).

Difficult situations can also arise in the reverse situation, when a relative feels obliged to undergo genetic testing so as to pass on the information obtained to others in the family, even when s/he would prefer not to be tested. This can arise in the context of familial breast cancer, for example, when testing the *BRCA1* and *BRCA2* genes in an affected individual might allow the family’s mutation to be identified. This may be very helpful for the at-risk relatives but the affected person, who already has a cancer, may not wish to face the prospect of the higher risk of a second breast cancer or of developing ovarian cancer (Hallowell et al. 2003; Foster et al. 2004). It may be difficult to determine whether a competent patient is ‘taking the interests of relatives into account’ in an appropriate way, as an expression of relational autonomy, or whether they are being subjected to ‘undue pressure’ by family members. The elucidation of the ethical considerations in this genetics context are interestingly different from the issues raised by family members who seek to ‘persuade’ a patient to make specific decisions about their own healthcare, when the interests of family members may differ (Ho 2008). Relational autonomy is of broad applicability to many genetic counselling contexts because it is a perspective from which all our lives are seen as socially embedded, and within which family relationships are seen as foundational to the rest. A strong version of relational autonomy also emphasises that our very ability to form human relationships arises out of our social origins and the care we all receive from infancy onwards (Scully 2008, pp. 160–163).

Other difficulties are faced by those patients in whom a sequence variant of uncertain significance (a VUS) has been found. How helpful will that be to relatives, even if their involvement might clarify the interpretation of the variant? (Vos et al. 2011).

In HD families, too, this sense of being pressed or even coerced by family pressure to be tested can arise when a young adult at 1 in 4 risk of the disease wishes to clarify their status while their at-risk parent prefers not to do so. The young adult may wish to make reproductive decisions in the light of their risk status while their parent, closer to the likely age of onset, may prefer to avoid confronting the possibility that they may soon develop the condition. Testing the young adult may reveal that their (so far unaffected) parent carries the HD gene expansion; it can be extremely difficult for such information to be kept secret within a family.

In such situations, we would encourage family members to meet to discuss the issues before any of the individuals are tested. Attending a genetic consultation together can sometimes be helpful in clarifying what is to be gained or lost by any particular course of action.

A thoughtful approach to weighing up the competing issues around personal genetic information in these and other contexts is set out in Inside Information (Human Genetics Commission 2002).

## Predictive genetic testing consent, competence and (non)directiveness

As discussed above, a predictive genetic test may be helpful in a clinical, medical sense because it allows optimal management of a patient at risk of an inherited disorder. When there is no medical intervention to recommend, however, the benefits of testing may be less clear so that considerations of the likely personal impact of test results on the individual and those close to them may be the key factor in whether to take the test. There may be a strong tension between wanting to know (that the result is favourable) and the fear of finding out (an unfavourable result). In those who know they are at risk of HD, fewer than 20% have a predictive test to see if they will become affected as, for many, the desire to resolve the uncertainty is outweighed by the wish to retain hope (that one will not be affected) (Tassicker et al. 2009; Baig et al. 2016).

In contrast to predictive testing for HD and most other neurodegenerative disorders, testing for many other conditions, such as the familial cancers and some of the inherited cardiac conditions, has the potential to confer some potential medical benefit. Once that becomes true for HD, the counselling in such contexts will change its nature completely and become much more like counselling for the *BRCA* genes or Lynch syndrome. Problems can arise in relation to predictive testing for cardiac- and cancer-related disorders when there is a mismatch between, on the one hand, how the preventive or therapeutic possibilities are presented by clinicians and understood by families in advance of testing and how, on the other hand, they are delivered by health services and experienced by families afterwards.

Constraints of space prevent a full exploration of predictive testing across the many different settings in which it occurs. Here we will focus on the context of testing for HD but we will mention just two other disorders where experience with predictive testing demonstrates the dificulties that can arise if the testing is approached as a straightforward, medically ‘obvious’ procedure, a matter for purely ‘clinical’ judgement, without attention to the counselling aspects of the decision. First, in the setting of hypertrophic cardiomyopathy (HCM), parents can arrange predictive genetic testing for their children and then regret their decision as the subsequent impact upon their children’s lives becomes apparent. Ethnographic studies that track families over some years are difficult to resource but can be very valuable (Geelen et al. 2011). In the very different context of predictive genetic testing for the Li-Fraumeni syndrome, a scheme for tumour surveillance has been proposed (Villani et al. 2011) but proof that it is of value at any age—and especially in childhood—is lacking. What stance should clinicians adopt towards predictive testing of young children? There is no single, clear answer (see discussion in British Society for Human Genetics 2010, page 21).

Returning to the non-therapeutic context of HD, perhaps the main question for the clinician is how firmly to challenge the patient’s request for testing. One does not want to be unhelpful and obstructive to those who wish to be tested but it is important to ensure that an irrevocable step is not taken before it has been considered carefully and from multiple perspectives. Predictive testing in the absence of any useful medical intervention—‘for information only’—is therefore one of the paradigmatic settings in which the ‘counselling’ aspects of genetic counselling come to the fore, and we therefore devote a considerable portion of this article to testing in this context, especially in relation to Huntington’s disease. HD was historically the first disorder for which predictive genetic testing became widespread and much experience has been gained in this setting. Testing for HD is also much commoner than for the other autosomal dominant Mendelian neurodegenerative disorders.

In this context of counselling for predictive testing without clear medical benefit, there is a blurring of the distinctions between factors to be considered by the professionals. There are clinical factors, issues of counselling and communication, and ethical considerations. These three categories merge and become very difficult to keep distinct. In this section, therefore, we discuss the clinical process in some detail.

Here, we will consider three more focused questions:

(i) How to respond to those who request testing but are unwilling to engage in the counselling?
(ii) How to respond to requests for testing made by adolescents and young people (ages 16–24). Can it be ***appropriately*** paternalistic to seek to delay the making of decisions by at least some patients?
(iii) How can we challenge patients—gently, with consideration and respect—to help them arrive at their decision in a more integrated or authentic manner?

We wish to influence not the decisions made by our patients but the way in which they make their decisions, so that we can all be more confident that taking the test—if that is what they choose to do—will on balance be helpful, whatever the result.

### Talking about testing—with Huntington’s disease as a model condition

Some individuals at risk of HD come to clinic convinced that they should have testing at once, without any discussion, and that they will be able to deal with whatever results emerge. After all, it is their ‘right’.

When it is explained that there is no medical benefit from testing and that we have a professional obligation to ensure that they have given valid consent before being tested, then most of these individuals agree to go through with the counselling process as recommended by international guidelines (MacLeod et al. 2013). Even if they feel they are humouring or tolerating us professionals, they will usually be willing to oblige. If they are not willing to do this, we sometimes fear that that their hold on calm consideration is fragile and likely to dissolve. Taking short cuts because of pressure to test rapidly is usually not helpful, although (very occasionally) it may be appropriate. It may, for example, be appropriate to accelerate the predictive testing process in a pregnancy. Even then, however, caution is important. Our experience leads us to believe that rushed testing in a pregnancy can have a grave and continuing impact on the individuals involved, including the next generation.

While the best practice guidelines cited above draw on evidence and experience, it is difficult to claim that they are fully justified on the evidence. Indeed, it would be difficult to know what evidence could be provided to confirm that a particular tradition of clinical practice was the optimum approach. Longitudinal studies could demonstrate that a certain proportion of the patients were adequately satisfied (especially those given a favourable result), but some patients would inevitably drop out from the study (especially those given an unfavourable result). Furthermore, it would be difficult to compare the outcomes of one clinical approach with those of another, if they differed substantially, as many clinicians would be unwilling to follow the full range of available policies. We, for instance, would not be willing to test those who request a predictive test without any counselling: we feel a responsibility to be as sure as we can be that everyone tested understands the range of possible test results and has had a real opportunity to reflect on the consequences, for them and others, for each result. In addition, evidence on some points could only be gathered by performing tests that we would consider unethical, and then following up those involved for several decades: as with the predictive testing of young children for HD. This topic will be explored further in the ‘Genetic testing of (young) children’ section.

In what follows, therefore, we present a composite account of our preferred clinical approach (even when it is supported by our experience rather than a body of research evidence) along with a discussion of some of the relevant research.

When a patient finds it emotionally difficult to engage with the discussion, it can be very helpful to reassure them that the decision is theirs and that we will carry out the test if they persist in requesting it. This can help overcome the sense that they must always present a strong, competent face to us in case our suspicion of their ambivalence or weakness means that we will deny them the test. In fact, of course, we feel much readier to test someone who can honestly discuss their ambivalence than someone who cannot admit such feelings even to her- or him-self, but an honest reassurance that we will really give away control completely can remove an important block that prevents some patients from engaging with the counselling. This reassurance, of course, should not be given if we in fact have reservations, as when we sense the features of depression or another mental illness.

An imbalance of power is an inevitable part of a medical consultation—if the professionals did not have greater knowledge and skill, then there would be no point in people seeking our services—but the imbalance can be destructive and, in these circumstances, we should work to minimise it. At the same time, we can acknowledge that the patient will be expert in their own situation and we need to work with them in partnership to achieve the best outcome possible.

When a patient appears unable or reluctant to engage with the counselling around genetic testing, the question may arise as to their ability to give consent to testing. In addition to having the information needed to make a decision and to not being subject to coercion (either blatant coercion or more subtle emotional manipulation), they should have the cognitive capacity to consent. For the professionals to have confidence that a patient has the capacity to consent under English law, professionals may need to weigh up this question: ‘Does this patient have a disturbance or impairment of the mind or brain and, if so, can this patient understand and retain and weigh up and communicate the information needed to make this decision?’ If the answer is ‘No’, then a decision about testing may need to be taken after discussion at a ‘best interests’ meeting. If there are real doubts about the patient’s capacity, or about their best interests, then a process of assessment may be required, potentially involving the courts or a legally appointed advocate.

When a competent patient argues that s/he should have testing without any discussion, and cannot be persuaded that the professionals have the responsibility to comply with standards of practice, an impasse may be reached. This is unusual but does—infrequently—occur. In such circumstances, the professional’s responsibility to provide good quality care may take precedence over the patient’s wishes. This can be framed as the professional over-riding the patient’s autonomy, but is perfectly legitimate in its own terms (Lantos et al. 2011). However, there is a separate discussion to be had about whether such a patient’s stated wishes do express their autonomous decision. From a Kantian perspective, which sets a high standard as to what counts as autonomy, this could be contested if the patient has failed to seek full information or has not given due weight to relevant and important considerations.

Our clinical approach follows that recommended in the consensus policy (MacLeod et al. 2013). We almost always have at least two meetings with the at-risk patient, sometimes more, with an interval of a month or so between each meeting. A genetic counsellor usually has a preliminary meeting to gather background information about the at-risk patient and their family, to seek confirmation of the precise diagnosis in an affected relative, to identify the wishes and expectations of the patient and to explain to them how the clinic functions. In the next consultation, we would usually discuss how they became aware of their risk of HD and how long ago. If this is recent, within the last year, that may be a reason to proceed more slowly with the testing. We would wish to know what they have learned about HD, through experience or in other ways, and perhaps provide additional information about the variability in age of onset and in the pattern of disease. Crucially, we will ask what it is that has triggered the request for testing *now* rather than last year or next year.

We would ask the patient how they feel they would cope with an adverse result, although of course no-one can be sure about this, and whether there is anything they or we could do in advance to help prepare for a period of real distress. It may be helpful to consider how they have coped with distress in the past, whether they rely on alcohol or ‘street drugs’, and whether they have a history of psychiatric disease or psychotropic medication. Such factors need not block access to testing but do help them to reflect on their request and whether it may be better to defer testing until they feel more resilient, their circumstances are more stable or they are better supported.

Other topics to discuss in advance of testing include who knows about their risk of HD (in the family, among their friends, their employer, etc.) and who knows that they are coming to clinic to discuss testing. To whom would they pass on news of their result? If they have children, are the children aware of their own risk? If the children are adolescent or adult, have they discussed the decision to be tested with them? If not, we would usually encourage them to do so, especially if a major reason for testing is so that they can tell their children. If an at-risk adult defers any discussion with their at-risk children (whether still minors or already adult) until their own risk is known, there is a trade-off between difficult scenarios. Either they must discuss the question of HD with their children when the children are still at 1 in 4 risk (when some of these difficult discussions will prove to have been unnecessary) or they must have a still more difficult discussion when their own decision to have predictive testing may have placed their children at 1 in 2 risk before they (the children) have any knowledge of their potential risk. We would generally suggest that the discussion is to be preferred earlier, as we have often seen the latter approach lead to resentment in the children at having been kept in the dark for too long. Breaking the family secret about HD at that point—once the child has been put at 1 in 2 risk—may also prove too difficult for some parents, who are still adjusting to their own positive result. This then leads to further secrecy within the family and a greater potential for emotionally destructive disclosures in the future.

This question of an at-risk parent discussing with their children—or other important family members—their own decision in advance of the testing is one of the points about which gentle challenging may be appropriate. Indeed, the impact of the test on the family as a system is a useful focus for discussion (Sobel and Cowan 2000). The question of ‘challenging’ about the impact on the family also arises in the context of testing those at 1 in 4 prior risk of disease (Maat-Kievit et al. 1999).

Following these discussions, the testing would then usually take place at a third appointment. This allows the patient some further opportunity for reflection and also for other questions to be raised, especially if the patient’s companion in the clinic—often a partner or spouse—has less knowledge and experience of HD.

An adverse result will often cause distress but may nevertheless be very helpful. However, how does the patient think that the quality of her life will be altered by this knowledge, between the test result and the onset of disease? In many ways, this is the crucial question for someone at risk to consider. It must be disentangled from the practical question of coping with the disease once it has begun. In addition, and also very practically, what might the implications of testing be for employment or career, for health and life insurance, and for driving? These topics are the aspects of HD most likely to trigger institutional discrimination and this often arises as a topic of discussion in the clinic (Erwin et al. 2010). Fortunately, in many countries, those at risk of HD now have a degree of legal protection, although that does not prevent stigmatisaton at the level of personal behaviour and intimate relationships in HD and other neurodegenerative disorders (Bombard et al. 2012; Mendes et al. 2017).

In the event of a favourable result, some patients whose burden of risk is lifted from them may nevertheless experience some very negative feelings (Huggins et al. 1992). They may feel excluded from their family, as no longer sharing in this key aspect of family life, and may find it hard to communicate with those siblings still at risk. Others may regret decisions they have made in the past (concerning marriage, career, reproduction). Such possible consequences of a ‘good’ result may be unanticipated by many if they are not raised in the discussion before testing.

Explaining the limitations of testing is also most important: the possibility of a grey zone result; the possibility of a misleading result if molecular confirmation of the diagnosis in the family has not been achieved because no sample of DNA is available from an affected family member; and the impossibility of predicting with any accuracy when or how the condition might begin to manifest. This can lead into a discussion of the unexpected outcomes of testing outlined above, especially the often unanticipated negative impact of a favourable result.

Having gestured towards the complexity of factors to be considered in coming to a decision about testing, we can now return to the patient who is reluctant to engage in the discussion. Some who want the test without the talk have clearly brittle defences, so that it would be irresponsible to test them at first meeting. Others, however, find it difficult to consider the hypothetical scenarios (‘How would you feel if ….?’) that comprise the core of pre-test counselling (Sarangi et al. 2005). This may be expressed as, ‘I’ll deal with it when it happens’. This may be a question of personality, of coping style, of psychological defence, of cognitive limitation, or of a failure of the professional and patient jointly to establish a connection—a bond of trust. Whatever the reason, the failure to engage can obstruct the counselling. This, on its own, is not a reason for refusing to perform the test, however, but an indication to the professional that an adverse result in such a patient may lead to greater distress as there has been less opportunity for them to rehearse and prepare for an adverse outcome.

While a reluctance to engage with the genetic counselling process will often need to be discussed and to become a topic within the consultation, there may be other, practical barriers that should be considered carefully, especially if they could result in social or geograhical inequity of access to genetic sevices for HD (as for other disorders). Thus, in countries with sparse and dispersed populations, geography may be a major barrier to accessing such services (Hawkins et al. 2013). Creative solutions may then be very appropriate, such as outreach clinics or tele-consultations, as long as the quality of service provided does not suffer. Simply providing the predictive test without the usual high (we would hope) standard of counselling and support would not be professionally acceptable.

### Patients who are ‘young’

As experience of predictive genetic testing for HD and other disorders ‘without treatment’ has accumulated over two decades, there has been a greater professional willingness to consider requests for testing from young people. While the international guidelines have suggested deferring a request for testing until at least 18 years but providing information, counselling and support to those younger than 18 years (MacLeod et al. 2013), others point out that there is no evidence of harm from testing children or young people (Michie 1996) and advocate for making predictive testing available to those younger than 18 (Duncan et al. 2008; Mand et al. 2013). However, the absence of evidence of harm is far from being evidence of the absence of harm. Consideration about the nature of the evidence that would be required to establish that harm had occurred is required; it may be impractical to gather such evidence, especially if the timescale involved might extend to decades.

Any age cut-off for predictive testing will be arbitrary, and this could indeed be counter-productive if those who cross the age limit then assume that they can have a test on request and without discussion. An age cut-off, however, may serve a useful function of protecting some very vulnerable individuals. Researchers who are enthusiasts for testing younger individuals sometimes report their research findings on those tested for a variety of disorders without distinguishing carefully between the conditions. Disorders with very different implications may be reported together, where some do and others do not have useful medical interventions. Down-playing these differences is unhelpful, and so is the lack of emphasis on the bias, among those tested for HD, who are willing to participate in follow-up research interviews towards those who test negative (a bias not so apparent for other disorders, where testing is clinically indicated). Clearly distinguishing tests for HD from those where the test is clinically recommended is crucial as the context of the decision to test is so different. There is a more general discussion about the genetic testing of children, and several of the international policy guidelines, in the ‘Genetic testing of (young) children’ section.

For the practitioner, there are competing tensions. We do not want to deny young people the potential benefits of testing, whether the test result is negative or positive, but nor do we want a young person to make a decision that they may soon regret. While at least some young people at risk of HD have an excellent understanding of the condition in context, others have been impacted by difficult family circumstances and their ability to make a good decision or their family’s ability to provide support afterwards may be impaired. Professionals will be concerned that factors such as denial, an adolescent sense of invulnerability linked to risk taking, an impulse to rebellion and a desire to separate from their family may all lead to the hasty making of decisions, if the young person has a limited ability to imagine the full range of possible test outcomes and how they may play out in their lives.

Some young people simply ‘wish to know’ without there being a specific decision that depends upon the result. We would suggest caution in such circumstances as the wish to know might apply only to the wish to have a good result. It may be difficult for any 18 year old to imagine their life at 30 years. How will decisions about relationships, courtship and reproduction work out in the light of different test results, as compared with a decision not to be tested (for the moment)? When and how will they raise the question of HD for discussion with a (potential) partner? Will it be easier to do that in the knowledge of their test result, or will that make it more difficult? On the other hand, they may be able to adapt well to their long-term prognosis at this age, as it may seem remote. Further research in this area, involving patient support groups and the accumulation of longitudinal case series, will be important.

Very helpful research into the experiences of young people at risk of HD draws attention both to the potential benefits of testing and the challenging circumstances faced by some young people (Forrest Keenan et al. 2015). Testing can be empowering and support the development of the young person’s identity. Factors that can complicate discussions with young people about testing for HD include the lack of family support that many experience, often indeed their sense of isolation. The response to test results can be especially difficult if the young person has only recently become aware of their risk or if the result differs from what they had expected. The impact of testing on subsequent family relationships may also throw up unanticipated difficulties.

In striving to develop a constructive approach to predictive testing in young people, it is very helpful to draw upon frameworks for understanding the normal processes of personality development. McConkie-Rosell and Spiridigliozzi (2004) set out a helpful framework while Richards (2006) examines the needs of adolescents in relation to genetic testing. Binedell et al. (1996) set out an approach to assessing the ‘maturity’ of adolescents seeking testing for HD. Some very helpful reports of testing young people for HD and other disorders have appeared, giving constructive suggestions as to how the genetic counselling process may provide improved support for this group. Thus Gaff et al. (2006) reflect upon their experience of genetic testing for familial bowel cancer in adolescents, while MacLeod et al. (2014) report the accounts and suggestions of young people after the process of testing for several different disorders (including HD) had been completed. There are good grounds for taking the patient’s age into account, through being especially open to flexibility with adolescents, but also being concerned to ensure that a request for testing is based on a considered and sustained wish, that the patient has been helped to think through the potential implications for relationships within the family, that the support available to the patient has been addressed carefully, and that the testing is not being sought as an act of defiance or self-assertion or as an automatic rite of passage for the 18th birthday.

#### Active non-directiveness

Returning to ‘non-directiveness’, it is necessary to distinguish the professional-patient relationship from a commercial transaction with a customer. If ‘non-directiveness’ simply meant doing what the customer requested, then, in our opinion, that would be an unacceptably shallow conception and a failure to fulfil our role. Simply following the patient’s instructions could amount to the professional acting on the basis of a patient’s whim or their ill-considered initial impulse in a difficult personal situation, which would be an abandonment by the professional of not only that particular patient but also their professional responsibilities. Rather than this, a practitioner has to provide the relevant information in an accessible way and raise factors for consideration that the patient may have not yet considered, as set out above.

Non-directiveness, rather contrary to its label, aspires to be an active process rather than a passive state. It can be difficult and challenging for the professional as well as for the patient; it demands full engagement with both the facts and interactionally with the patient in the consultation. There are many decisions about genetic testing that are much more routine because there is a clear medical recommendation to make, as in testing for certain susceptibilities to malignancy (Elwyn et al. 2000). It is still good practice to discuss the emotions that may be faced in those different circumstances but the intent in raising such issues is not to challenge a patient’s decision whether to be tested but to help improve their preparation for facing the test results. In contrast, in the context of HD, one is wanting to engage the patient much more in thinking//feeling her way through the decision and, potentially, to change her mind. We would want to help her to imagine her potential responses to the different outcome scenarios, so that she can effectively self-select as to whether she feels strong enough to go ahead with testing or not (yet).

In essence, active non-directiveness recognises that professionals will influence their patients, and that a process of influence is actually desirable. One can then work with this positively rather than simply denying or attempting to minimise any influence, as a shallow non-directiveness would do. Training for the genetic counsellor will aim to develop insight about the influence that is exerted and how to make this influence appropriate and helpful, to enhance the decision making process of the patient (Kessler 1992; Wolff and Jung 1995; Clarke 1997). This approach can be framed in the language of counselling or psychotherapy, or it can be viewed as grounded within a philosophical tradition. John Stuart Mill’s sense of autonomy incorporates questioning and challenging that promotes the other person’s autonomy (discussed in Lantos et al. 2011), but it can also very readily be considered from within a Kantian perspective or from within virtue ethics.

In the next section, we will move from predictive genetic testing to the question of pregnancy and prenatal diagnostic testing. There is a strong continuity between these two sections as the prenatal context is the other setting within clinical genetics in which the question of non-directiveness has particular salience and force.

## Prenatal diagnosis, prenatal screening and disability

The use of genetic testing in various forms of prenatal diagnosis raises many ethical issues. These include questions about whether, and under what circumstances, it can be acceptable or even the best course of action to terminate a pregnancy. Here, we will focus on the questions that arise when the **selective** termination of pregnancy is being considered. Given that a woman and her partner wish to have a baby, under what circumstances is it acceptable or proper (even ‘to be recommended’) to investigate the embryo or foetus so as to make a decision about whether to continue the pregnancy or to end it, and perhaps try again. The key question is the process of deciding between a current conception and possible future conceptions: which are to be accepted and welcomed, and which are to be rejected and terminated?

Here, we consider this by looking at three subsidiary questions:

(i) How can prenatal screening or diagnosis be offered without the offer in itself conveying a strong recommendation (to accept the test and terminate the pregnancy, if the foetus is affected)? Is this best approached from the perspective of population health? Or should we concentrate our attention and efforts on implementing an actively non-directive approach in prenatal clinic discussions? Furthermore, what are the social conditions under which antenatal screening for genetic conditions could be offered without the social circumstances being, in effect, coercive?
(ii) How do we maintain respect for those affected by genetic conditions and congenital defects while offering couples such reproductive decisions?
(iii) What difference will non-invasive prenatal testing (whether diagnostic or screening) and population screening for recessive disease carrier status make (a) to the experience of pregnancy? and (b) to the experience of having an affected child?

### Does offer = recommendation?

In the setting of the routine antenatal clinic, in which the offer of antenatal screening is made and considered, there are many processes that could routinise patient participation and thereby undermine the achievement of non-directiveness. Some of these processes depend upon the personalities and motivations of the professionals involved, but many will be impersonal and more structural, relating to clinic processes rather than the behaviour of individual al staff. It may require hard, active work on the part of staff to make it apparent to patients that they are being offered a screening programme and that they need to consider it carefully before climbing aboard the conveyor belt (Clarke 1991). However, the language used by practitioners may exert a strong influence which they (we) should learn to check and control. Examples of language use that needs to be watched (and challenged) include ‘This test is to provide you with reassurance’ and ‘Most people do this …’ (Pilnick 2002). These statements can be uttered in many different ways and with quite contrary intentions but they may be understood (‘received’) as being coercive, with the intention of directing patients to accept the ‘offer’ of routinised screening programmes.

In this context, it is helpful to reflect on the differences between the three main approaches to ethics. While the consequentialists look at the outcomes of reproductive decisions—often adopting a perspective from which they are confident in knowing a good decision when they see one—others prefer to be guided by principles (especially the autonomy of the pregnant woman), while virtue ethicists consider what it means to be a good (enough) parent or professional, and what decisions or actions such a parent or professional would be likely to make or to undertake.

Savulescu and Kahne (2009) urge us all to bring into this world the best quality infants that we can and to use all available technology to achieve this. Judgements about the ‘quality’ of infants, however, may be both highly contested and insuperably complex; is a foetus with cystic fibrosis one to be avoided? (Boslett 2011) There are questions of disease severity and treability to be considered. In addition, the contemporary focus on rights, especially the autonomy of the pregnant woman, could lead to a supine acceptance of any request made by a woman or couple but could also lead to discussions about the place of women in society, which we will return to in a later section. If women experience their lives as a burden because they are powerless in a patriarchy, does that make it acceptable to collude with the patriarchy and terminate female foetuses so as to save them from the same difficult lives as their oppressed mothers have lived? Finally, at what point can or does a woman become unconditionally committed to her foetus or child? Such commitment is regarded as virtuous, as an integral part of the nurturing that a good mother will provide (McDougall 2007). But does this begin at conception? at quickening? at birth? or, as in some ancient societies, once the child has survived the high mortality of early childhood? Or, in some more contemporary circumstances, the checks on ‘foetal quality’?

The consideration of a mother’s commitment to her infant reminds one of a mother’s deferred commitment to her foetus that Katz (1986) described so eloquently as a consequence of the introduction of antenatal screening by amniocentesis: commitment to the foetus//baby was delayed until the results of chromosome testing were available. This deferral of commitment, however, came at a social and personal cost. The very experience of choice about participation in screening can be experienced as a burden (van Berkel and van der Weele 1999) and it is difficult, although perhaps not impossible, for this choice to be offered in a ‘neutral’ or non-directive fashion (Clarke 1991; Rapp 2000; Williams et al. 2002). In the routine antenatal clinic, discussions about screening for genetic disorders often concentrate on the facts and not on the meaning and weight of the decision to be made and its implications (Hodgson et al. 2010). The difficulty of the decisions that mothers are asked to make has been explored in several studies. The notion that the pregnant woman may feel a conflict between different ethical principles in coming to her decision may not be the most helpful way to understand her position. Instead, it may be recognised that she experiences a conflict of interests between her foetus and other members of the family, perhaps especially her other children (García et al. 2009).

For a couple to terminate a wanted pregnancy is always going to be difficult and distressing. The long-term sequelae of such terminations on the grounds of foetal abnormality have not been much studied and the work of van Mourik remains valuable (White-van Mourik et al. 1992; White-van Mourik 1994). The support needs of the women and couples involved are substantial and the recognition that the mother sometimes has the moral role of taking on herself the suffering of ‘the child who would have been born’ can be important.

A singularly helpful study of the decisions made by pregnant women (and their partners) about antenatal screening, as well as other types of genetic testing, is that of Scully et al. (2007), in which it is shown how women will often make a series of microdecisions in a pregnancy, deferring any important decision to the last possible moment. It can be too difficult and challenging to confront their situation in its full complexity and so they may focus on only what is immediately in front of them. It will not usually be our role to confront those who respond to events in this way in any but the most gentle fashion.

The situation of women considering prenatal diagnosis because of a known high risk of disease in their foetus, arising from a family history of a rare disease, is very different from the experience of women in routine antenatal screening. The complex and agonising decisions to be made when a prospective parent is at risk of Huntington’s disease, for example, are well recognised (Downing 2005; Klitzman et al. 2007).

When we turn to a population focus on a single disease, and look at the collective consequences of decisions made by many individuals, it becomes clear how difficult it can be to decide when a collectivist attempt to give families genetic information and choices becomes an inappropriate attempt to lead them to make specific decisions. Thus, the paper by Helderman-van den Ende et al. (2013) on pregnancies at risk of Duchenne muscular dystrophy (DMD) shows that many female carriers who could have been identified as carriers in advance of the pregnancy had not in fact been identified. Using a package of measures, including mutation testing on chorionic biopsies found to be female at prenatal diagnoses for risk of DMD and more effective family cascade testing, it would be possible to recognise more female carriers before they have affected sons. These carriers could then be offered the opportunity to have genetic counselling. The stated concern in this paper is to achieve the prevention of more cases of DMD, while fully respecting the rights of the women involved to make their own decisions. What perspective should one adopt on this issue? Is the population-level concern appropriate, or is it likely in practice to prove coercive, or at least manipulative and directive? If we assert that genetic services are merely working to ensure that prospective parents are able to make informed reproductive decisions, is our response adequate or are we evading the moral question at the heart of this issue?

One recommendation of the paper (Helderman-van den Ende et al. 2013) would be to adopt a different approach to the genetic testing of children for carrier status. If we do this, are we being eugenicist, i.e. protecting ‘Society’ from the consequences of decisions made by family members who have not faced up to their responsibilities, whether the decisions are made actively or by default? Or are we simply improving the services available to DMD families? How should we discuss and evaluate the cumulative population consequences of multiple decisions made by individuals?

In the context of countries that are poorer and less developed, how do the arguments play out in relation to genetic testing or population screening for carriers of beta-thalassaemia? (see also the “Ethical issues in our multi-cultural UK society” section).

### Respect

Does it devalue an individual with a genetic condition if society chooses to establish antenatal screening programmes to reduce the numbers of individuals born with the same condition? Do they have a right to object that this is disrespectful and promotes discrimination against them? This concern has been labelled the expressivist objection to antenatal screening programmes for genetic disorders.

One approach to this is to talk with those affected by Down syndrome, for example, and see how the question of antenatal screening ‘for’ (‘*against*’, *perhaps?*) their condition impacts on them. As anyway feeling devalued, it is no surprise that antenatal screening can add to their sadness (Alderson 2001; Barter et al. 2017). This does not give affected individuals a veto over such policy questions (Shakespeare 1998), but it certainly reinforces the need to be as sensitive and respectful as possible in the implementation of such a programme and the need to take great care in producing information about screening. What is one to say about Down syndrome to inform prospective parents who have no personal experience of individuals with the condition? How can information be provided in a ‘balanced’ manner, when one is proposing to eliminate such individuals? (Hippman et al. 2012). The ‘twice-told tales’ element—the difference in information provided during pregnancy or after birth—was pointed out by Lippman and Wilfond (1992) and applies as much today as ever before. If we shift the provision of information to the preconception setting, could that give prospective parents a chance to reflect more calmly and come to a decision with which they can subsequently feel confident (Schoonen et al. 2012)?

If we shift to consider disorders for which prenatal diagnosis may be sought by those with a family history, but which are not generally included in antenatal screening, how are the considerations different? What can we learn from reflecting on them? In contrast to population screening programmes, there will often be much less need to provide information about the disorder, as it will often be well known to members of the family. However, the factors that shape the decisions about reproduction may have no less impact on affected members of the family, who may be greatly saddened that a close relative—a sister or brother, a son or daughter—has decided not to risk having a child like they are. This is not the invariable response—some affected individuals would encourage relatives not to transmit their condition—but it is a possible response. It can be thought of as the ‘expressivist objection’ to antenatal screening but at a much more personal and intimate level, within the family. This has been studied recently within families affected by X-linked hypohidrotic ectodermal dysplasia (XHED) (Clarke 2013, 2016) and spinal muscular atrophy (Boardman 2014a, b) and it is to be noted that the different modes of inheritance (sex-linked and autosomal recessive, respectively) alter the dynamics of decision-making within families.

One aspect of coping with a genetic disorder, which also often influences the making of reproductive decisions, concerns the stigma attached to the specific condition. In XHED, for example, the stigmatisation that affects many affected males can be a major determinant of a female carrier’s decisions about bearing children. She will often have seen her male relatives struggle with the stigma and cope with varying degrees of success. This may be at least as important a factor in her decisions as the physical and clinical, medical problems of life as an affected male. One wonders how this plays out in other disorders, where there is a combination of physical anomalies with unusual appearance and also, perhaps, some cognitive impairment. Which aspects of these disorders is most important for the affected person and for his or her relatives? How major a contribution is made by the prospect of stigmatisation? One study that gives some insight into the strength of feeling around these issues is the interview-based study of Kelly (2009), which shows how the parents of children with serious disorders often prefer to avoid making decisions about prenatal diagnosis that would cause inner conflict; they may avoid conceiving, or avoid prenatal diagnosis, or at least avoid terminations of pregnancy. They choose not to make choices.

Would we be comfortable with a decision about prenatal diagnosis and the termination of an affected pregnancy on the grounds of ‘the cost of care’? If we mean the cost of care borne by the state, then we are back in the days of state-sponsored eugenics. However, in these times of state retrenchment and public austerity, what about concerns by families that they would find it difficult to fund the facilities that their affected child would be likely to require? One example put forward has also related to XHED, where the costs of dental care may be substantial (if dental implants are preferred to the much cheaper dentures)? (Aldred et al. 2003). To paraphrase, concisely but perhaps unfairly: ‘The termination costs less than dental implants and we would not want our child to make do with dentures’. How do we respond to that?

More generally, we can consider how—in what terms—to discuss normality and disability. This maps across onto the concepts of social inclusion or exclusion, normalisation or marginalisation. There is a rich literature here that deserves to be explored by those of us in clinical genetics, whose daily work involves us in using but often not examining the concepts of disability and normality (Bridgens 2009). This exploration can usefully involve developing personal relationships with disability rights groups to explore the issues, as well as a purely theoretical engagement (Peterson 2012). Engagement with the experience of those living with impairments can bring valuable insights, both through personal relationships and from second-hand encounters in the literature. Important studies and narrative accounts are available of life with numerous genetic conditions, such as achondroplasia (Ablon 1984; Adelson 2005), neurofibromatosis type 1 (Ablon 2013) and HD, including the dimension of life with the risk of HD (Konrad 2005; Browner and Preloran 2010). The accounts of parents whose children have neurodevelopmental difficulties from infancy, for whatever reason, have demonstrated the recalibration of expectations of their child and of their role as parent (Voysey 1975; Landsman 1998, 2003) that is familiar also in ‘quality of life’ assessments of adults with a range of impairments: those affected are much less dissatisfied with their lives than ‘objective observers’ think would be appropriate (Scully 2008). The implications of this can be read in different ways. This question of how to read such different perspectives is at the heart of the disability rights movement, which is not prepared to concede that the lives of disabled individuals are not as worthwhile as those of anyone else.

Studies of illness that are grounded in a phenomenological perspective are bringing deep insights but have usually addressed acquired or later-onset disease (e.g. Carel 2016) rather than congenital disorders manifest from birth: phenomenological studies that address these conditions would be most valuable.

The field of disability studies has flourished for some decades. This has led to the firmer underpinning of ‘advocacy’ and a strong link between the experience of disability and its analysis within academia (Parens and Asch 2000; Ablon op cit). There has been debate about whether it makes sense to speak of a collective disability identity, or whether the different types of disability and impairment are too disparate for this to be coherent (Scully 2008). This includes efforts to anchor the experience of physical and sensory impairment in the sociology of the body. The field of disability studies has also retained its sense of the social roots of disability and the context from within which disablement arises (McLaughlin et al. 2008), and has absorbed much from feminist theory (Woodin 2012). Through activism and advocacy, it has also contributed to the welfare of many with motor impairments, so that the facilities for wheelchair users in the UK have improved very substantially over the past four decades. The representation of the experiences of those with cognitive impairment is less developed; those with severe cognitive impairment may be especially vulnerable to having their status as fully human persons undermined (Edwards 2006).

One particular area in which further empirical investigation and conceptual clarification would be expected to be immensely helpful is that of the disorders of sexual development and gender assignment decisions in infancy, where ‘treatment’ and even surgery may be driven by parents’ confusion and their understandable distress but where such pressures may in the long run work to the detriment of the patient (Feder and Karkazis 2008).

### Reproduction into the future

Finally, in this section, we turn to consider some of the new technologies and how their application to reproduction may change the experience of pregnancy and raise new issues or raise familiar issues but more powerfully.

With the advent of non-invasive prenatal testing (NIPT) through the sequencing of cell-free DNA in the maternal plasma, a modest fraction of which derives from the foetus (technically, the chorion), several questions arise. At present, women who are reluctant to undergo any sort of prenatal genetic screening can explain that they wish to protect the pregnancy from the risk of miscarriage associated with invasive procedures. This account of why she declines a screening test may be used to justify herself to the professionals or to critics within the family. The advent of NIPT undermines this ‘excuse’ and may make it harder for her to sustain her avoidance of testing. To what extent this is an important consideration—how widespread this practice has been—is unknown but it is clear that NIPT has the potential to become even more routinised than the current types of antenatal screening, with consequences for the validity of consent for such tests (van den Heuvel et al. 2010).

While NIPT may complicate the decisions of those reluctant to use prenatal diagnosis, it will in other ways bring some very clear benefits in that fewer miscarriages are likely to result from invasive procedures as fewer will be performed. NIPT can operate as either a first-tier screening test or an intermediate tier (between the first tier of serum-plus-nuchal-translucency screening and invasive procedures). NIPT is substantially more sensitive and specific than current first tier screening so there will be many fewer amniocenteses and fewer miscarriages. However, it is important to remember that the positive predictive value (PPV) of NIPT for Down syndrome (DS) varies with the chance of DS in that pregnancy and is far short of 100%; for a standard risk pregnancy (not already known to be at higher risk) the PPV is about 80%; as a second tier test, its PPV is at least 90% (Taylor-Phillips et al. 2016; Nuffield Council 2017).

If a majority of pregnant women continue to use antenatal screening, the proportion of DS foetuses that are identified through screening and then terminated will increase, so the number of infants with DS at birth will decrease. How will this be experienced by current and future persons with DS? And by their families and the professionals who care for and support them? There will certainly be some sadness (Skotko 2009). How do we place a value on this, when we see DS as another way of being human rather than a disorder? This is where the question of ‘balance’ in representation becomes so important but, ultimately, cannot be achieved. On the one hand, DS may be seen as a ‘disorder’, as it can have grave medical consequences as well as sometimes a very severe impact on cognitive development. On the other hand, many with DS experience fulfilling lives and make a positive contribution to the lives of those around them. The problem is that the different ways in which DS is being examined may be incommensurable.

As screening by NIPT is implemented more widely, the range of disorders it is being used to identify is also broadening out to include many recognised chromosome microdeletion syndromes. The decision as to what conditions to include in screening seems at present to be driven largely by considerations of marketing, with companies seeking to claim that their NIPT test “covers more” than their competitors. However, the performance of the test changes as new conditions are added to the testing package, and the PPV for additional tests is usually lower than for DS, so that the advantage of NIPT over other approaches to screening for the autosomal trisomies may be lost: the number of invasive tests performed, and so also the number of procedure-related miscarriages, would both increase. These and related issues are considered with care in a recent report (Nuffield Council 2017).

What other conditions may be included in screening, beyond chromosome deletions? Women should be aware that incidental findings may emerge, including (rarely) evidence of malignancy. Will parents pay to find out about traits or predispositions for which there may be no clinical utility (and would therefore not be included in evidence-based health care)? It may also be possible to assess the foetus for non-medical traits or for adult-onset disorders, for which it would usually be regarded as inappropriate to test young children (Deans et al. 2014). Where should society draw limits as to what is permitted, or should this be left to each individual in the marketplace?

Foetal sex is another trait that parents may be interested to determine early in a pregnancy. While superficially harmless, this may be used to terminate pregnancies carrying a female foetus in societies that devalue women. Indeed, late terminations of female pregnancies (after ultrasound scans) have very substantially skewed the sex ratio at birth in parts of India and China. As an act of solidarity, we believe that foetal sex should not be determined by NIPT except when it is clinically relevant in relation to a sex-linked disease (Nuffield Council 2017).

The combination of NIPT and carrier detection by high-throughput DNA sequencing has the potential to eliminate not only Down syndrome but also, in western countries and wealthy countries elsewhere, all chromosome copy number anomalies (at least all recognised pathogenic deletions and duplications) and virtually all cases of autosomal recessive disease (Edwards et al. 2015; Human Genetics Commission 2011). There would clearly be difficulties with the implementation of such a programme, especially the problem of sequence variants of uncertain pathogenicity that are detected in autosomal recessive loci. However, there would in addition be profound consequences if ‘society’ were either to make a collective decision to prevent the birth of people affected by these conditions, or allow social pressures to work to the same end for those who could afford the technology. Society in general, and health care in particular, would look very different. The argument against screening would lead us to remain in our present situation, where prenatal diagnosis to avoid the birth of a child with a serious recessive disorder is only available once a couple has had at least one affected child. Should prenatal diagnosis and the selective termination of an affected foetus only be available under those circumstances? (Why?)

This brave new future, with few children affected by chromosomal or autosomal recessive disorders, could be represented either as a major triumph or as an inhuman dystopia, in which something vital has been lost. How are we to make up our minds?

Another challenge that will soon arrive in the clinic is that of rational, gene-based therapies that can be applied in utero. The circumstances in which such treatments may be preferred to the current use of prenatal diagnosis and the selective termination of affected pregnancies will have to be considered and defined. Cost will be a major issue: will private or state-backed health insurance schemes or national health care schemes be willing to provide such treatments if they are both costly and of uncertain or incomplete effectiveness? (The field is reviewed from a multidisciplinary perspective in Schmitz et al. 2018).

## Genetic testing of (young) children

We have already discussed some of the issues that are raised by the genetic testing of young people in the ‘Predictive genetic testing consent, competence and (non)directiveness’ section. Here, we turn to consider decisions about genetic testing of young children, who are unable to participate in the discussions and decisions about this. What genetic information is it appropriate to generate about young children?

We take for granted that diagnostic testing is (almost) always appropriate, if the cause is being sought for a clinical condition currently affecting a patient of any age. When a sick child is being investigated, it may be wise to raise in advance with the parents the possibility that the disorder may be genetic, perhaps with familial implications, but that is not a reason for not performing the investigation. What must be considered is when it would be appropriate to perform tests of no relevance to the child before s/he reaches maturity. This includes predictive genetic testing for adult-onset disorders and carrier testing for autosomal recessive disorders, or for sex-linked disorders and chromosome rearrangements.

While reflecting on this, there are some subsidiary questions to consider.

- How do children find out about the problem in their family?
- What are the factors to be considered in relation to the predictive testing of young children for an adult-onset condition, or carrier status testing for any disorder, when the condition is already known to run in their family (when the child is too young to participate in the decision-making)?
- Does the weight of these arguments change when incidental findings emerge from testing a young child, of which the family would otherwise be unaware?
- When is it reasonable to test a young child as a preliminary to arranging their adoption?

### Finding out

Perhaps the first question to consider, before thinking about genetic testing, is how children come to find out about a genetic condition in the family. In discussing with parents in clinic ‘how to tell the children’, it would be common to encourage open disclosure but in a gentle and age-appropriate manner, in contrast to the idea of keeping the problem secret and then planning a disclosure session as a single, discrete event. Such an event could be daunting for the parent and emotionally traumatic for the child, if they are old enough to appreciate what is being said. The decision not to mislead or deceive but to give the information sought by a child over time, as they mature, is a policy that is simple to recommend but there have been few studies of how parents do in fact communicate difficult genetic information to their children.

The first study to address this issue is probably that of Manjoney and McKegnay (1978), which described the cycles of non- or mis-communication between generations in families affected by polycystic kidney disease. A failure to pass on information in a timely and supportive manner condemns the next generation to discover their diagnosis in the same difficult, and sometimes catastrophic, fashion as their parent had done. A few studies have reported the experience of communication within families affected by Huntington’s disease. Holt (2006) reported the experience of two contrasting families, and came down strongly in favour of a drip-drip trickle of information: a gentle, supported and slow-paced disclosure of information to children. Etchegary (2006) reported a typology of patterns of discovery, with four trajectories of finding out: (i) ‘something’ is wrong; (ii) out of the blue, (iii) knowing but dismissing, and (iv) growing up with HD. It is the period of ignorance before finding out that shapes the account given by the ‘child’ (perhaps by now an adult) about their recognition of their situation of risk.

One large study has looked at these issues in the context of several disorders and found that children prefer the slow and gentle drip feed of information over time (Metcalfe et al. 2011). Further research in this area, in other disease contexts, would be important and worthwhile.

#### Current policy

Numerous professional bodies have recommended caution, at least, in performing genetic tests on children unless it is clearly a diagnostic test (to explain a problem affecting the child now) or is needed to arrange treatment, prevention or health surveillance during childhood (British Society for Human Genetics 2010; Borry et al. 2009 for the European Society of Human Genetics ESHG; Botkin et al. (2015) for the American Society of Human Genetics ASHG; and Ross et al. 2013 for the American Academy of Pediatrics AAP and the American College of Medical Genetics and Genomics ACMG). These position statements are very similar, except that the AAP/ACMG statement, but not the ASHG policy, has been disappointingly weakened from its previous joint position with the ASHG from 1995. These more cautious policies are based on two considerations (i) that it is important to preserve the autonomy of the child so that, as a future adult, she is able to make her own decision about testing. This in effect applies one understanding of Feinberg’s concept of an Open Future to this area of competing rights and interests, and (ii) that testing the child as a child results in the loss of privacy of their test result and may harm the child if it influences parental or institutional behaviour towards her, with scope for (perhaps subtle) discrimination between siblings whose test results differ.

A libertarian or an enthusiast for testing children might argue that such decisions should be left to parents, who often make proxy decisions for their children, and this decision should be treated as no different. The drawback of such an approach is that many parents will want to know the genetic status of their child for any disorder that runs in the family. Once they have tested the child, that information will be known within the family but it removes the possibility of the child playing a key part in discovering the information. This may be especially important if adjustment to an unfavourable result is substantially better when the at-risk individual has made their own active decision about testing as a ‘mature enough’ adult and has not had testing imposed on them when younger.

The shift in policy between the previous ASHG/ACMG policy and the current AAP/ACMG document (Ross et al. 2013) is contained in this sentence, ‘After careful genetic counselling, it may be ethically acceptable to proceed with predictive genetic testing to resolve disabling parental anxiety or to support life-planning decisions that parents sincerely believe to be in the child’s best interests’. This could be seen as an invitation to any parents focused on their ‘right to know their child’s genetics’ to claim that they are disabled by anxiety, or that they need to take the child’s genetic status into account in making important decisions (such as, presumably, investment in their education or health). It is unclear to us that testing would in fact be justified under such circumstances and this policy formulation appears too open to abuse, especially in a country with a largely private system of health care.

In one ‘ideal’ scenario, information that is not clinically indicated or useful until the child is mature would not be generated until then. The ‘child’—by then a Young Person or perhaps a mature adult—makes the decision to be tested. Their involvement in the decision, with appropriate counselling support, gives them a choice and makes it easier for them to accept an adverse result whereas, for a parent to give them an adverse result generated years before may be very difficult for the parent and may lead to anger, resentment, disbelief or rejection on the part of the ‘child’. The testing will have been performed in childhood at the request of the parent(s) and for their own reasons. The child’s interests have been overridden by the parents’ (often misplaced) concern or by curiosity labelled as “disabling anxiety”. If the professionals comply with the inappropriate parental request, they are very likely to have been negligent of the best interests of the child. This sets up problems for the future, with perhaps a failure ever to disclose the result to the child, or a disclosure made in difficult, perhaps damaging, circumstances.

We know that carrier information generated about young children or a foetus is often not transmitted to the child (Jolly et al. 1998; Jarvinen et al. 1999); the same question has not been studied in relation to ‘clinically inappropriate’ predictive testing, but our experience of inappropriate parental requests leads us to believe that they are likely to find it even more difficult to pass on that type of information.

Our ‘ideal scenario’ has the advantage of demonstrating trust in the child to make a wise decision about testing, which creates an opportunity for personal growth in both parent and child.

The case against testing a child without a clear medical indication will not always be so strong. In carrier testing for autosomal recessive disease there may be much less at stake than in predictive testing for HD. If families can accept such carrier information and pass it to their children in an open and constructive way, then much may be gained and little lost by the early testing (Vears and Metcalfe 2015). In testing for carriers of sex-linked and chromosomal disorders, however, the chance of unhelpful consequences is greater, such as the stigmatisation of carriers and altered expectations of their future roles and relationships. Such problems are still more likely to occur in late-onset dominant disorders where there is no health benefit from testing in childhood. Professional discussion of many varied scenarios is presented in Arribas-Ayllon et al. (2009) and Parker (2012). Problems may arise when family loyalty to an affected sibling is in tension with the welfare of future children, as with carrier testing in the unaffected sibs of those with X-linked disease (James et al. 2003) or autosomal recessive disease (Fanos and Johnson 1995a, b).

Other difficulties arise with predictive genetic testing if it is difficult for the parents or the children to live with the results. Although they have existed for some years, we still find some predictive information difficult to handle and to explain to parents. Tests that indicate a substantial risk of behaviour problems in childhood and of schizophrenia in adolescence and adult life are very difficult for parents to manage (Hercher and Bruenner 2008). How does one respond to the difficult behaviour of a child who (one fears) may be destined to develop psychosis? Does one give in to all challenges so as not to cause frustration and resentment? Or adopt a ‘zero tolerance’ approach of very firm boundaries? Or try to treat the child as if one had not been given this information? And if your child is found to be at risk of sudden cardiac death (Hendriks et al. 2005), how does one decide what pattern of exercise and social life to recommend? And how can this be enforced? As discussed in the ‘Predictive genetic testing consent, competence and (non)directiveness’ section 3, some families in which children were tested for their cardiomyopathy have regretted the decision to arrange the testing (Geelen et al. 2011). It will be important for researchers to build long-term relationships with families in which these issues arise if we are to gain insight into responses to different patterns of professional practice.

### Incidental findings

An additional consideration about testing children for late-onset genetic disorders has arisen in the setting of genome-wide testing, especially exome and whole genome sequencing. The incidental identification of a (probably) pathogenic variant, likely to lead to a late-onset disorder such as cardiac disease or a malignancy, may occur in a child who is being tested to determine the cause of a different condition, perhaps a complex neurodevelopmental disorder. If the family has so far been unaware of this risk, then this incidental recognition of a disorder may, in some circumstances, be very helpful as one of the parents is likely to carry the same pathogenic variant. Recognising this may be to the parent’s great clinical benefit, and hence to the benefit of the child too.

This approach was proposed by a working group of the American College of Medical Genetics and Genomics (ACMG), being proposed as a requirement for any genome sequencing of children, whether in research or diagnosis (Green et al. 2013). A list of 56 genes was to be examined with the disclosure of pathogenic variants, whether or not any prior consent had been obtained. This policy was subsequently revised by the ACMG Board in 2014 with the concession that disclosure should not be required without prior discussion and parental consent, which brings the ACMG recommendations more into line with those of other bodies such as the ESHG.

While the ACMG policy has some strong arguments to recommend it, the provocative wording of the initial policy and the rush to see it implemented betrays the True Believer enthusiasm of some members of the working group. There is a vision of a person’s genome sequence being available throughout their life as a resource for use in making any health care decision. However, there are both technical and ethical problems with this. What we accept as a genome sequence will not be the same in 10 or even 5 years as is current today and the practicalities of data storage are not simple, so that repeating the genome sequencing when required may be a better solution than long term data storage with its difficulties of perpetually needing to update the IT systems, both software and hardware, and of ensuring privacy and data security (Chadwick et al. 2013; Clarke 2014). There are also political difficulties with this model of genetics in health care, including the inappropriate individualisation of health policy and the neglect of equity. Despite these quibbles about the details, however, the ACMG report did set out a convincing case that the incidental recognition of a late-onset genetic disorder in a child should generally be disclosed to the family, unless it is known that the family is already aware of the problem identified. A decision not to seek genetic testing by one who knows s/he is at risk is altogether different from their failure to seek testing because they are ignorant of the risk.

#### Adoption

When we turn to adoption, there are other challenges. Health professionals are usually reluctant to carry out genetic testing on a child being considered for adoption if the test would not usually be performed on that child under different social circumstances. In contrast, those from a social work background may see the long-term advantages to the child of being adopted as crucial to the child’s future. If that takes one or two additional genetic tests, then a successful adoption would be well worth the price. How do we resolve this difference in perspective, when both paediatricians and social workers are involved in the adoption process?

The restrictive view of genetic testing in adoption has been put forward by several authors (Morris et al. 1988; Newson and Leonard 2010) and challenged by others, who see that the interests of a child may indeed be bound up in the outcome of a decision about adoption (Jansen and Ross 2001). It has also been argued that genetic testing, even a genome sequence, may be appropriate in children being considered for adoption because the family history information available to the prospective adopters is often poor and the genome sequence will compensate for this (May et al. 2015). Counter arguments to this last claim are numerous and persuasive, for the present, as the interpretation of a genome sequence is still far from straightforward and the introduction of yet more genomic uncertainty into the portfolio of information about the child is unlikely to help the prospects of her or him being adopted.

The debate so far has largely related to information about a known risk, most often Huntington’s disease (HD). While health professionals may be more open to persuasion in relation to genetic testing for some cancer predispositions, such flexibility is much less likely in relation to HD given the small number of those at risk who seek testing as adults, the great potential for stigmatisation and discrimination, the altered expectations likely to be held by parents about the child with an adverse result, and fear for the child’s future (including the prospects for being adopted) if s/he tests positive. Improving prospects for treatment may alter this but, as yet, they are still too remote and uncertain.

The force of the ‘ideal’ setting discussed above is still applicable in the setting of a possible adoption, with the implication being that the ideal adoptive parents are those who can accept that the child has a risk of future disease but are willing to adopt in any case, without insisting on certainty either way. If there were an excess of prospective adopters, then a ‘Judgement of Solomon’ solution might apply. Whether or not that surfeit of adopters is to be found for any particular child will vary between communities and countries.

The other setting in which genetic tests are considered in relation to adoption is chromosome evaluation for neurodevelopmental difficulties, with the method now in use being array comparative genomic hybridisation (aCGH). This has largely replaced chromosome analysis but the paediatricians engaged in adoption work are still often finding (at least in the UK) that the greater diagnostic yield of aCGH is accompanied by a challenging injection of uncertainties from the variants of uncertain significance (VUSs) in the result and, less often, the incidental findings of disease risk for some other problem in the birth family. Of these difficulties of interpretation, it is the VUSs that impact on the adoption process. Prospective adoptive parents may be willing to adopt a child with known learning difficulties, whether or not there is an explanation apparent on chromosome studies, but less willing to accept a child where there is a cloud of uncertainty around the interpretation of the aCGH report.

One can foresee the time when genome-wide investigation of children to be adopted might become standard practice, at least in some jurisdictions, although we would prefer to continue without generating such clinically inappropriate datasets. The notion that genomic investigation will compensate for the lack of a detailed family history in a child being adopted is not scientifically plausible and certainly not a convincing reason to override the usual considerations of medical ethics. Furthermore, if genomic investigations are performed on a child being considered for adoption, or already adopted, a plan must be in place as to how to pass potentially important findings to the birth family, if they arise.

## High-throughput testing for genetic disorders

High-throughput investigations have transformed biology as a science. Instead of experimental design for the testing of hypotheses, the frontier of research is now the development of tools for data interpretation: making sense from the data. The same change has impacted on clinical investigations: it is no longer so difficult to generate sequence information, but it can be very challenging to make useful diagnostic sense of it for the individual patient. The history of human laboratory genetics was the focusing onto progressively smaller details—starting with the chromosome and working through linkage studies with close markers, to the specific gene and then its sequence, looking for point mutations. Over the last decade, however, that level of detail has become available across the whole genome at a single step, the whole genome sequence (WGS).

Given this fundamental shift in the methods of laboratory genetics (Clarke et al. 2012), it is now necessary to choose what information to attend to and to pass along the chain from laboratory scientist to bioinformaticist to clinician, and then further, to the patient and family. While it is possible to pass the entire WGS to the patient as a data file, this would miss the point: patients look to health professionals for more than that. They want and require an interpretation and recommendations that are supported by the available evidence.

What difficulties are raised by these new circumstances? The two major problems to address have already been met above: (i) variants of uncertain significance (VUSs) and (ii) incidental findings (IFs), also known by other terms such as ‘off-target’, ‘additional’ or ‘secondary’ findings.

### Incidental findings and variants of uncertain significance

VUSs are not entirely new to genetics, of course. They have been recognised for as long as laboratory genetics has existed, but it is the scale of the problem that is new. The principal current approach to VUSs is that advocated by Berg et al. (2013) and subsequently adapted by ACMG (Richards et al. 2015) of a systematic approach to classifying each VUS as being more or less likely to result in disease, using five ‘bins’ to one of which each variant is assigned (not pathogenic; unlikely to be pathogenic; of uncertain pathogenicity; likely pathogenic; definitely pathogenic). The grounds on which these assignments are made are well recognised and will not be rehearsed here except to note that surprises can occur: a likely pathogenic variant may be reassigned to the ‘definitely not pathogenic’ bin, as new information and experience accumulates, or a variant thought likely not to be pathogenic may turn out to be pathogenic. The process of making these assignments is described by Timmermans (2015) in a study that examines the trust placed by laboratory scientists in their laboratory and bioinformatic processes and in each other and in their clinicial colleagues. They then make judgements as to which variants, in which genes, should be reported to the clinician who has requested the investigation. The clinician will then pass to the patient or parents the clinically important, ‘definite’ results that are relevant to the patient’s problem, but then has to decide when to report any of the other results, especially if a VUS has been found in a gene thought likely to be relevant to the disease in the family. If this VUS may be pathogenic and further investigation may clarify this, then of course it must be disclosed, although that will sometimes lead to families misunderstanding this; they may believe that the VUS must be pathogenic and act on that misunderstanding.

The variants found may be sequence variants or copy number variants (CNVs). CNVs are detected by array comparative genomic hybridisation (aCGH) as well as by sequencing methods, especially WGS (Boone et al. 2013). There is more experience with reporting CNVs to families than sequence variants but they still generate many difficulties for both the clinician and the patient or family: how does one ‘act on’ or understand a CNV result that indicates a modest susceptibility to attention deficit disorder, autistic traits, schizophrenia or intellectual disability? Information has to be imparted in a very tentative manner to help prevent families jumping to unwarranted conclusions.

Addressing only the question of IFs, and restricting this to only the 56 genes that ACMG initially recommended for assessment for possible IFs, one recent study found that the mean number of likely pathogenic variants was 1.69 per patient (Jurgens et al. 2015). Broadening out to WGS as a whole, there are even more serious problems of interpretation. The level of uncertainty of interpretation of many of the variants found when performing WGS on healthy volunteers, the incomplete sequencing coverage (even of known disease genes), and the inadequate knowledge base for deciding when to trigger further diagnostic investigations (Dewey et al. 2014) suggest that WGS may be best regarded as a research investigation rather than a regular clinical tool, except as a last resort in the face of serious disease without a diagnosis, and when the clinician has made her best efforts using currently established methods.

### Consent and Recontacting

When explaining WGS, aCGH, clinical exome analysis or even a large gene panel to a patient or family, it is essential that the issues of VUSs and IFs are discussed. Without that, valid consent cannot have been given to the investigation. However, it is here important to take a short detour into the question of consent more generally. ‘Taking consent’ and, ‘giving’ it are the two sides of one activity, a social encounter. What is going on for the two (or more) participants in this encounter? The professional will want to put on a convincing performance as a competent, caring and ethical professional but, at the same time, s/he will want to ‘get the box ticked’ and move on to the next task. Similarly, the patient or parent will (usually) wish to perform as a sensible and responsible patient or parent but may not want to read through pages of typescript in a small font and, in any case, may be too anxious really to consider the details of what is being proposed. This will often be a recipe for collusion between both parties to have a brief but not challenging or intense discussion, so that neither party has to dwell on it. They can hurry through the consent step as not much more than a formality. Further work is needed to examine ‘what is going on for the participants’ in the setting of consent for diagnostic genomics, along the lines of work by Corrigan (2003) on clinical trials and Shipman et al. (2014) on biobanking.

Consent for genomic investigations has to be ‘broad’ (Helgesson 2012). The line between diagnostic and research procedures may (understandably) be blurred—as in the 100,000 Genomes Project of NHS England - so the need to avoid the misunderstanding that participation in the research will have therapeutic value (i.e. the ‘therapeutic misconception’ familiar from drug trials) is clear (Halverson and Ross 2012).

The initial suggestion of ACMG (Green et al. 2013) that information be returned even without prior consent was flawed but useful in at least three ways: (i) it stimulated interest and debate, (ii) it set a defensible limit on the list of loci about which IFs should be returned, thereby reining in the potential competition between research groups and among commercial companies in what IFs would be sought and disclosed, and (iii) it drew attention to the difference between the usual ‘Genetic Testing of Children’ scenario, where there is a family history of a specific disorder, and this very different situation in which there is often no prior awareness of a genetic diagnosis in the family so that there could well be further casualties of the condition (perhaps the parents or other relatives) if the incidental finding were not acted upon. The detailed composition of the ACMG list can be debated, and has been revised (with four additions to, and one removal from, the list that now totals 59 genes: Kalia et al. 2017) but the principle has been established of reporting back to families if a clinically important and actionable IF is found for the benefit of the family as a whole (not only the child). Previously known disorders within a family are of course excluded from this, and disclosure of these additional findings to the parents must have been raised with them as part of the process of ‘information and consent’, so that they should have agreed to this possibility in advance. This respects the (contested) ‘right *not* to know’. However, the potential social costs of this ‘right’—in terms of *either* failing to respect considered personal decisions *or* denying their relatives access to potentially important health care—are clearly overwhelming. The case for exempting information of clinical utility generated about children from the ‘right not to know’ is very strong. A parental decision not to consider information of likely practical clinical benefit to their child, or perhaps to their parents or siblings, could hardly have been guided by the overall best interests of the others involved.

It will also be necessary to address two other areas in the consent process: (i) which results should be returned to the patient (or parents)? and (ii) when or how often should findings be reassessed? The question of what results to return has generated a lot of debate in the clinical and bioethics literatures. Research is simpler—the obligations on researchers are less complex—if only aggregated results are given back to participants (Beskow et al. 2012). Indeed, that was all that would have been sensible when GWAS results were at issue. With sequencing results (exome or WGS), however, the findings of each individual are of much greater potential significance. This alters the situation dramatically (Tabor et al. 2011; Kaye et al. 2014). The PHG Foundation (Hall et al. 2013) and Knoppers et al. (2014, 2015) have proposed helpful frameworks for those generating sequence information in the research setting.

In clinical practice, the process of consent for paediatric genome-wide investigation has been studied in several settings. Lessons have been learned about the minimum information that should be provided and discussed with parents (Burke and Clarke 2016). In a research setting in Toronto, the attitudes of the parents of children having genomic investigations for the purpose of a diagnostic assessment have been assessed and are of real interest (Anderson et al. 2017). The participants, a small and somewhat atypical subset of those whose children were being investigated in the hospital’s Genome Clinic, described their sense of having difficult and even distressing findings ‘inflicted’ on them but feeling unable to opt out of receiving these additional//incidental findings because of their sense of a burdensome duty to know all the potential problems that might face their child (an ‘inflicted ought’). In an insightful commentary, Newson reminds us that the parents’ response to IFs is specific to the setting of this particular clinic, where at least some parents felt that the unwanted information had indeed been inflicted upon them, when they were at best ambivalent about receiving it (Newson 2017). This arose out of the requirement to agree to receive IFs as a condition of accessing the diagnostic application of the WGS.

In relation to consent for genome investigations in children, the decisions will usually be made by their parents ‘in the child’s best interests’ (Ross, 2013). In the UK, there is no arbitrary line drawn at a specific age, before which it must be the parent who gives consent for medical investigations and treatments. Where a child of less than 16 years has some capacity to be involved in the discussion, this will be encouraged and they may give their views. The patient may be able to assent to medical procedures but the parent(s) will usually take responsibility for formally providing consent. Even young children can make important contributions to the decisions made about them, and research into consent to elective surgery in childhood has demonstrated this with great effect (Alderson 1993). Between 16 and 18 years, either the child or the parents can give consent to treatment. From the age of 18 years, a patient will be presumed to have the capacity to give consent unless there are grounds for considering that s/he may lack capacity, in which case their capacity has to be assessed. As mentioned earlier in the context of predictive testing, young people of less than 16 years can give consent, and in limited circumstances even refuse consent, as long as they can demonstrate adequate maturity of judgement. The essential test for those of less than 16 years is ‘Gillick competence’, as established by a court ruling under the Common Law of England & Wales, in which the test is whether the child has achieved, ‘sufficient understanding and intelligence to understand fully what is proposed’.

For adults, capacity is framed as the ability, with support if necessary, ‘to understand the information they are given; retain that information for long enough to make a decision; weigh up the information; and communicate their decision’. It is important to appreciate that capacity is not a single attribute but varies, being specific to the particular question at issue, and even fluctuating with the same person over time.

The question of when (and how often) to reinterpret any VUS results has also been widely discussed. With the rapidly increasing numbers of VUSs and the accumulation of information and experience about variants, the status of VUSs can change, with their status as pathogenic or benign usually becoming clearer. That, however, raises the question of how genetic services could periodically re-evaluate all the VUSs they have identified, and then how they could contact the patients whose variants had changed in a clinically meaningful fashion. What obligation has a service to take on this challenge? How can the costs of this additional service be met, when these are likely to escalate exponentially for a decade or more before any sort of stability of variant interpretation is reached? These issues remain challenging although constructive work to find solutions is in process. One difficulty with some proposals is that they rely on a pro-active engagement with the patients and families involved (Pyeritz 2011; Dheensa et al. 2017). This would certainly mean that only some of those who could benefit from ‘recontact’ will do so, is highly likely to be socially inequitable, and could well drive an increase in such inequity (Tudor Hart 1971).

What about investigations whose results are of little or no validity or applicability? The giving of information of no clinical utility, such as GWAS-based risk modifications, would *not* be regarded as equivalent to the disclosure of IFs. However, such results may be of interest in one way: if an increased risk of disease is assigned to the patient based on GWAS-related risks, does it lead to the modification of behaviour that would be recommended in the light of that genetic susceptibility? This type of personally assessed disease risk does not seem to lead to the ‘appropriate’ behaviour change: it does not tap into a strong, additional motivator to encourage compliance (Marteau et al. 2010; McBride et al. 2010). In any case, such ‘advice’ is consistent neither over time, as more or different SNPs are utilised, nor between test providers. What is of much more ethical concern is the inequity of access to genome-based and DNA sequencing-based investigations of proven value, which comes down to a question of the political system within which health care is being provided (McClellan et al. 2013).

An overview of the issues raised by the ‘genomic approach’ to clinical genetics is given by Clarke (2014), following a broader assessment of the science as well as the clinical implications (Clarke et al. 2012). A very helpful ethics-based framework for assessing the applicability of genome-based investigations in paediatric practice has been formulated by McCullough et al. (2015). A likewise constructive suggestion from Newson and colleagues is that genomic investigations should be approached in professional training and in discussions between patients and professionals as an exercise of uncertainty management: the ethos of genomics *is* uncertainty and this should be acknowledged and embraced (Newson et al. 2016).

#### Newborn metabolic screening and whole genome sequencing in the newborn and the foetus

As with the shift from focused to genome-wide investigations in laboratory genetics, biochemical screening of newborn infants for metabolic disorders has changed fundamentally with the introduction of tandem mass spectrometry (TMS). This has greatly increased the capacity of screening in terms of the volume of samples analysed in one laboratory and also the scope of what is screened for: TMS is able to identify a much wider range of different molecules than the previous, chromatography-based methods. This technological shift has greatly expanded the number of metabolic disorders that can be detected by screening.

This in turn makes it possible to identify newborns affected by (or destined to become affected by) disorders that do not fulfil the conventional, 1968 WHO criteria for screening devised by Wilson and Jungner. The key mismatch between these criteria and the new possibilities is that early diagnosis often fails to lead to improved outcomes for the infant because there is no effective treatment.

If the inclusion of a disorder in a newborn screening programme fails to meet the established criteria for screening, why might it nevertheless be a reasonable course of action? One response is that the parents become aware of the child’s diagnosis sooner than if they waited for the child to present with symptoms. They avoid the extended diagnostic process that might otherwise result, which can be an ordeal reminiscent of the Odyssey. They are spared this process, reported vividly in the case of Duchenne muscular dystrophy (Firth et al. 1983). The average time lag between the family seeking medical advice and achieving a diagnosis was 23 months in one study; we have no reason to think it has improved. Late diagnosis is a serious problem in many disorders (e.g. Bouwman et al. 2013).

The other major factor is that this earlier diagnosis enables parents to take account of the diagnosis in their plans for (further) children. They may then be able to make future reproductive decisions in the light of their risk of having affected children (Bombard et al. 2009). Newborn screening for DMD has shown that (most) families appreciate the early diagnosis, if they have made an active decision for the screen for DMD to be included in their infant’s newborn blood spot test (Parsons et al. 2002). This provides both the potential benefits: avoiding the distressing diagnostic Odyssey and enabling informed reproductive decisions. Will this also be true for other untreatable genetic disorders?

The question of whether to require specific and distinct consent for disorders like DMD or SMA (Swoboda 2010), for which treatments are still experimental and not yet routinely available, is important (Ross 2006; Ross and Clarke 2017). It would require a two-tier consent process for newborn screening, with the traditional screen being heavily recommended for the direct benefit of the infant and the second-level, opt-in, tests being for broader benefits to the family. It may be helpful to ‘tweak’ the information-and-consent process to emphasise the distinction between the two types of test (Parsons et al. 2000), thereby improving the quality of the consent and the satisfaction of patients and professionals. It may further be argued that a different set of criteria should be developed to help think through which disorders could legitimately be included in extended newborn screening (Petros 2012).

A further concern about the extended panel approach to newborn screening is that it identifies as patients many who are far from developing symptoms, some of whom will never become unwell (e.g. half of those with medium chain acyl coA dehydrogenase deficiency, MCADD). This leads to a new category of the ‘patient-in-waiting’ (Timmermans and Buchbinder 2010). There may be additional categories of patients where even early treatment for the affected child will not lead to a ‘cure’ but to a long and difficult illness; a family’s choices in future pregnancies may need to be based on a realistic assessment of treatment outcomes, which may not be available until long-term research programmes have reported their results. It should also be acknowledged that the natural history of some of these biochemical disorders has actually been clarified by newborn screening, which is an unbiased method of case ascertainment. The decision to include some disorders in screening may not always have met the Wilson and Jungner criterion of an ‘adequate’ knowledge of the natural history (Timmermans and Buchbinder 2012) but has led to an improved understanding of the penetrance of the condition.

Newborn screening may lead to the identification of other patients in the family, in addition to the diagnosed infant, perhaps an older sibling or a parent. A health care system that identifies such cases but fails to support the families in meeting the needs of these additional patients is clearly failing to discharge its responsibilities (Buchbinder and Timmermans 2011).

Another category of newborn screening which has been introduced, often for a genetic condition, is screening for impaired hearing. This gives access to cochlear implantation for those with severe deafness. In those for whom cochlear implants do not permit highly effective communication, the use of sign language may have been preferable. The decision to use sign language rather than cochlear implants may also be preferable to the community of The Deaf, which is formed around and through sign language. How will this community fare if so many potential recruits are now treated with implant surgery? How can we be confident that we have chosen the best treatment to be selected for each child?

Finally, in this section, we turn to consider WGS as a routine for newborn infants. Some useful diagnoses would emerge earlier than when waiting for a symptomatic presentation, although that is not always an advantage if it generates distress but there are no effective interventions to alter the natural history of the condition. This would enormously amplify the ‘patient-in-waiting’ category, i.e. the ‘worried well’ of asymptomatic individuals who have been found to carry sometimes pathogenic genetic variants in genes associated with late onset or incomplete penetrance. This would apply, for example, to those carrying a variant in a gene known to make a contribution to the risk of a cardiac dysrhythmia or cardiomyopathy. VUSs may also be found in such genes. The difficulties of VUS interpretation would loom large and the handling of information about adult-onset disorders and carrier status of largely reproductive significance. The disadvantages of WGS performed on newborn infants as a routine screen (i.e. unless used to achieve a diagnosis for a severe and complex condition) are serious (Howard et al. 2015).

It must not be forgotten that newborn screening by WGS could never replace the more traditional metabolic screen, both because the prognostic value of the metabolic screen has great power and because the commonest disorder identified, congenital hypothyroidism, often has no specific genetic cause (it most often arises from a failure of thyroid gland development and migration). Furthermore, this model of newborn WGS, both as a form of newborn screening and as a lifetime resource, depends on long-term data storage as already discussed, with the attendant difficulties and costs (Clarke 2014).

In the present state of our ability to interpret genome sequence data, the generation of WGS on newborns as a routine - rather than its infrequent use in attempting to achieve the diagnosis of rare and complex disorders, which would be its ‘obvious’ (clinically warranted) application—appears to be a wolf in sheep’s clothing: a massive research programme in the guise of a population screening programme. However, while it would be too vast to secure research funding, it could not possibly warrant funding as a population screening programme because it lacks any evidence of clinical utility and the opportunity costs would be immense. Given the extent of the unmet health needs of the population of the wealthiest country on the planet—the country from which the proposal for newborn screening by WGS emanate—we cannot imagine such a programme being justifiable in the foreseeable future, even if it were entirely benign in its consequences.

A recent proposal to go beyond neonatal to routine, unrestricted *prenatal* WGS by non-invasive prenatal testing (NIPWGS) appears to be a still less reasonable proposition, given that it addresses no specific clinical goal. The proponents appear oblivious to the confusion, anxiety and distress that would inevitably accompany its implementation (Chen and Wasserman 2017). While it is suggested that there would be an intensive programme of parental education about genome-based testing and an opportunity for moral reflection, there seems to be little awareness of the challenges this would entail at multiple levels. Furthermore, it is difficult to see what is driving the programme, or what it is hoping to achieve, apart from an increase in genome corporation profits at the expense of much potential family misery.

This proposal was accompanied in the same journal issue by a set of commentaries, some of which elaborated on the problems likely to be caused if this rash proposal were implemented, while others were supportive. The argument of Chen and Wasserman that they are not drawing arbitrary, ‘eugenic’ lines between foetuses, but instead empowering parents to make their own decisions, seems wilfully to ignore that this reproductive empowerment is taking the shape of consumerist eugenics, open to manipulation by Big Data Corporations, health insurers and fashion, and fits neatly into the account of contemporary ‘backdoor’ eugenics by Duster (2003).

Kaposy (2017) reminds us that the offer of choice in a pregnancy will not always be experienced as liberating but can be burdensome and disturbing, and that there is no call or demand for prenatal NIPWGS in apparently healthy pregnacies. (While it may be very possible for marketing agencies to create a demand, this would most likely be achieved by a re-framing of NIPWGS as the virtuous expression of parental responsibility and/or an enviable privilege of wealth). Furthermore, Kaposy (correctly) sees values as constructed and context-responsive rather than monolithic and fixed, so that the parents’ values may be moulded by the process of seeking information through foetal WGS. We might add that the process is likely both to reflect and to reinforce an inappropriately strong form of genetic determinism.

Ravitsky et al. (2017) reject the claim that NIPWGS would increase parental autonomy. They point out that the prior risk of disease in a healthy foetus is low and this impacts on the interpretation of the findings. In addition, the quantity of data that could be imparted is massive and the demands on members of the public to make sense of this are utterly unrealistic. The apparent belief of Chen and Wasserman that efficient information transfer and a limited period of reflection will allow couples to march happily on with their lives suggests that we encounter very different people in our clinics from those they meet. We do not recognise in their fantasy the world that we inhabit. As Mazersky and Sankar (2017) explain, the making of decisions is in reality far removed from the rationalistic account of Chen and Wasserman. The direction of inference from genotype to phenotype is an entirely different sport from explanation of phenotype in terms of genotype, as indicated by Botkin et al. (2017), Chen and Wasserman seem grossly to exaggerate the capacity of the former. Botkin et al. also make the point that the troubling aspects of the selective termination of pregnancies on the basis of prenatal diagnosis are not removed but exacerbated by extending the range of ‘abnormalities’ considered to be acceptable grounds for the termination of an otherwise wanted pregnancy, especially when the genotype=>phenotype link may be insecure. We are not writing from an anti-abortion position fixed in principle but from a recognition of the human pain and suffering involved in the termination of wanted (and perhaps perfectly healthy) pregnancies.

Munthe (2017) makes the additional point that Chen and Wasserman ignore the distinction between the technology perhaps having some acceptable applications, while at the same time being completely unacceptable as a population screening programme.

Some of the supporters of NIPWGS focus attention away from the foetal decisions being made to the management of information about the future child (if s/he survives to term). There are two particular problems with this. First, the fact that NIPWGS would of necessity be offered during pregnancy means that it cannot be aimed primarily at post-natal interventions for the benefit of the child, as WGS would be simpler and cheaper in the neonatal period or later in childhood. The proposal of Chen & Wasserman must therefore be intended primarily to lead to decisions about terminating apparently healthy pregnancies; otherwise, why not wait? The other possible explanation - aiming at treatments applicable in utero—is still premature even for most of the malformations that may be amenable to maternal-foetal surgery; treatments in utero for genetic disorders are still more remote. Secondly, the argument that it should be up to parents to decide whether to generate ‘difficult’ predictive information about neurodegenerative disease in their children (Rhodes 2017) ignores the fact that most adults at high risk of such conditions choose not to find out, and that this is for very good reasons given the realities of human psychology and family relationships. (They may not be pure, autonomous Kantians but can still be good people!)

## Ethical issues in our multi-cultural UK society

How does one address ‘culture’ or ‘cultural difference’ in genetic counselling? Like Odysseus, one has to steer one’s craft between two waiting perils, the Scylla of cultural stereotypes and false homogenisation on the one side and the Charybdis of vain attempts to be blind to cultural difference on the other. Avoiding both errors is important and requires insight. This is a matter for training in counselling skills, personal development and supervision rather than chapters on ethics; we do not have space to treat these aspects further here. But some topics can—must—be broached.

One of the settings that most often raises ‘culture’ as an issue in the context of genetic counselling in Britain is consanguinity. What does the professional need to be aware of when addressing this? First, that the topic is, or may be, highly charged. Within the UK, many of those from communities in which consanguineous marriage is customary and often preferred will have been treated badly by health professionals, and perhaps others, when this topic has been raised in the past. If they have a child with autosomal recessive disease, they may have been blamed for this, perhaps quite explicitly, and may even have been insulted. More likely, however, the blame will have been implied, in the tone of voice perhaps. Such behaviour by professionals is unacceptable and unprofessional. While it may occur less frequently than in the past, we believe that it still happens all too often.

In the UK, at least, the sense of blame may have been compounded by statements from Members of Parliament and public health officials, who sometimes talk about consanguinity as a major social problem, especially among Muslims drawn from South Asian populations, and suggest that this community should change its long-standing practices. (They tend not to mention other communities that favour this practice). The consanguineous family may have felt that their community was under assault for this mark of difference, as well as for other religious and political factors. It should not be forgotten that the anti-consanguinity message of the health professionals can find a target elsewhere too. There are other consanguineous marriages in the UK, both within other ethnic minorities and also within the white British majority. Such statements can impact on their people’s (often already strong) sense of shame and guilt for any genetic problem in their family.

From the perspective of the community practising consanguineous marriage, it will usually be seen as entirely natural and a force for stability in the family, which enhances the status of women and which is not usually associated with genetic disorders in the community’s children. Professionals must not forget the positive features of consanguinity and should not use genetics as a route through which to voice cultural prejudice. Furthermore, it is unhelpful, unfeeling and unprofessional to blame parents for having children with a serious inherited condition. The incidence of recessive disorders is increased in consanguineous families and communities, and needs to be raised in a respectful manner in any discussion about reproductive risks, but the global context within which such marriages constitute a widespread social tradition must not be forgotten (Modell and Darr 2002; Hamamy et al. 2011; Bittles 2013).

Moving to consider lay and folk beliefs about the causes of malformation and other birth defects, a wide-ranging review of such beliefs by Kenen (1980) is enlightening. It shows the recurrent patterns in such beliefs from around the world and reminds us that these have been common in Europe until quite recently; superstition may still play a role in the thinking of people from many parts of the world. The Urdu-speaking community from South Asia resident in UK has a wide range of beliefs about birth defects and other paediatric conditions that fit into Kenen’s scheme, and a range of attitudes to the use of genetic investigations and, in pregnancies, to the use of antenatal screening that might lead to the offer of a termination of pregnancy (Shaw and Hurst 2008; Bryant et al. 2011; Shaw 2012). It is most important to remember that ‘religion’, whether Christianity, Islam or any other, is not monolithic and homogeneous but diverse and often negotiable. Professionals must make no assumptions about how a patient thinks or how they will respond in a given circumstance simply because they come from a specific community or religious group. Many of the attitudes and practices in minority ethnic groups are shared with those from the majority white British ethnic group.

Particular solutions have sometimes been devised to meet the needs of specific communities, whom one might expect to be reluctant to use some aspects of genetic technology. Some Orthodox Jewish communities use an anonymous programme of carrier screening with the results disclosed to the matchmaker (not to the couple) indicating that a particular couple would not make a good match (i.e. both are carriers of the same autosomal recessive disorder) (Raz and Vizner 2008). This approach may lead western clinicians to feel horror at the loss of autonomy this entails but, in the context, it may at least allow such communities to begin to engage with this aspect of modern health care. However, it is important for the ‘western’ genetic counsellor (i.e. from a western family and community background) to appreciate that many from ethnic minority groups will have their own views that are shaped by their experience of disease in the family and community as much as by the beliefs and values of their religion (Atkin et al. 2008). Make no assumptions!

When consanguinity is preferred within a community, it is possible to maintain this custom but reduce the risks of serious genetic disorders. One effective approach is to marry more distant cousins. Another approach is to make carrier screening available, either before marriage or in the antenatal clinic. A number of countries in North Africa and the Persian//Arab Gulf are using next generation sequencing to define the recessive gene mutations present in their populations, often unique to small population isolates or endogamous groups within a country. Schemes differ in whether each individual tested is given their own results and whether the results influence decisions about marriage or decisions about pregnancy, prenatal diagnosis and children. They also differ in whether they are mandatory or optional, and the extent of the pressure to comply with screening.

Several countries have developed a range of compulsory interventions with the stated goal of reducing the birth incidence of genetic disorders. One approach has been to carry out tests for carrier state of genetic disorders, sometimes of specific disorders such as beta-thalassaemia, either before marriage or conception or in the antenatal clinic. A different approach in China entails a (fairly) set and structured approach to assessment of a married couple. The relevant law has changed recently but before that the potential parents were judged on criteria familiar from Nazi Eugenics, with an interview and examination aimed to support the doctor making decisions about the desirability of two persons having children together (Hesketh 2003).

Infertility and childlessness are problematic within all societies, and the form taken by this problem will of course be shaped by the culture and its expectations. In the lives of South Asians who have settled in UK, there may be a tug between the influence of tradition and the influence of the individuals’ new experiences and acculturation into local life (Hampshire et al. 2012). Secrecy around infertility and its investigation and treatment is common in many communities. Infertility seems to be particularly wounding to the male ego, perhaps from some conflation of infertility and impotence. Adoption can also be a very difficult idea to accept within some communities, so that neither the admission of infertility nor adoption as a constructive ‘remedy’ may be recognised as an available option for the infertile couple (Bharadwaj 2003, a study set in India). This of course increases the pressure on couples to use infertility services—but in secret.

Another potential conflict of perspectives arises in relation to the preference for sons that is stronger within some communities than others (although it was very powerful in UK until not so long ago). The sex ratio at birth is heavily distorted in some Indian states and in parts of China, with the sex ratio at birth being around 900 females per 1000 male births across India and below 800 females per 1000 males in some states (Madan and Breuning 2014). A comparable distortion of the sex ratio is also found within China and can be seen in some western countries with communities of South Asian origin when there is no legislation to restrict foetal sex selection. The long-term social consequences of such ‘unbalanced’ sex ratios are unpredictable and may change over time but will surely generate major social problems. This imbalance of the sexes results in both India and China from the widespread (although illegal) use of medical technology to determine foetal sex and terminate the female foetuses, and is reinforced in India by the (also illegal) system of dowry payments. The same would doubtless have happened in UK in previous centuries if the technology had been available (i.e. if witchcraft, magic or prayer had been more effective). In UK, where foetal sex selection is not permitted, professionals can become concerned when the talk or behaviour of patients in clinic suggests that the family may be motivated to choose a ‘social’ termination of pregnancy after foetal sexing performed as part of prenatal diagnosis for other reasons. Foetal sex selection has been shown to occur in some South Asian groups resident in Canada (Urquia et al. 2016).

Our western and feminist response to this scenario is horror at the systematic devaluation of women that this implies. Where women from communities that practice foetal sex selection support this approach, this adds to the horror as it supports the sense that life is hard for the women in these communities. A contrasting feminist response has also been proposed, reflecting a position of support for women’s decisions even when a rejection of sex selection and strong support for women’s position in society would seem more constructive (Moazam (2004). The sense that women’s social status needs support in some of these societies is reinforced by a sad tale of stigmatisation within UK of a woman of Bangladeshi origin who was affected by neurofibromatosis type 1 (Rozario 2007). There are also unsettling parallels between foetal sex selection and the low social status of women in some communities, on the one hand, and antenatal screening ‘for’ Down syndrome and the social status of affected individuals in many western countries on the other (Alderson 2001).

Decisions about what disorders are sufficiently serious to warrant a termination of pregnancy have a cross-cultural dimension. However, it would clearly be difficult to disentangle the many factors that may contribute to this. Thus the stage of economic development within a society, confidence in support from the family, community or state, intrinsic beliefs about what makes life worthwhile and the moral status of the embryo are all important but unquantifiable and often negotiable factors. One example is the striking diversity of views about termination of pregnancy for deafness, with the lack of confidence of support from the community in raising a child with deafness given as one factor that may contribute to a decision to terminate in some countries (Nahar et al. 2013). For a child born into a Deaf family, of course, rearing a Deaf child may be the preferred option and sign language will often be chosen over cochlear implantation. Personal experiences and the degree of acculturation are likely also to be relevant factors.

## Conclusion

We present this whistle-stop tour through ‘the ethics of genetics in medicine’ as an educational resource. It has evolved since the turn of the century and will doubtless continue to change and develop as the issues that arise in our clinical practice also change. We have only been able to cover some aspects of this broad topic but we hope that the general approach adopted here proves useful and that it can be adapted to the particular circumstances in which you are working.
